# Draper‐ATG3 Interaction Positively Regulates Autophagy to Mediate Silk Gland Degradation in *Bombyx mori*

**DOI:** 10.1002/advs.202504664

**Published:** 2025-09-15

**Authors:** Shiyu Zou, Yuhan Luo, Yue Jin, Wenhui Jing, Yuxin Huang, Yanting Liang, Yinghui Li, Zhihua Hao, Yusong Xu, Huabing Wang

**Affiliations:** ^1^ College of Animal Sciences Zhejiang University Hangzhou 310058 China

**Keywords:** ATG3, autophagy, *Bombyx mori*, draper, metamorphosis, silk gland

## Abstract

Autophagy critically regulates developmental cell death during insect metamorphosis, yet the regulatory mechanisms underlying autophagy‐dependent cell death remain poorly defined. Here, the engulfment receptor Draper's essential role in tissue clearance and remodeling is reported. Initially, it is shown that Draper is evolutionarily conserved in most insect species. CRISPR/Cas9‐mediated knockout in the lepidopteran model *Bombyx mori* demonstrates that Draper deficiency impairs autophagy activation and delays middle silk gland degradation during metamorphosis, while its overexpression enhances autophagy induction. Proteomic profiling reveals that loss of Draper disrupts silk protein metabolism, ubiquitin signaling, and autophagic substrate degradation. Through liquid chromatography–tandem mass spectrometry and coimmunoprecipitation, a direct Draper–autophagy‐related protein 3 (ATG3) interaction is identified, which enhances autophagic activity. These findings bridge a critical knowledge gap in how developmental signals mechanistically engage core autophagy machinery to ensure precise tissue remodeling. This study redefines autophagy initiation paradigms by identifying Draper as an evolutionarily conserved regulator, providing a unified framework integrating developmental timing, phagocyte signaling, and metabolic clearance in metamorphosis.

## Graphical abstract

Draper is evolutionarily conserved across most insect species. Loss of Draper inhibits autophagy activation, delaying middle silk gland degradation in the silkworm during metamorphosis. Draper interacts with autophagy ‐ related protein 3 (ATG3) to orchestrate autophagosome formation—a process essential for silk gland degradation during the larval‐pupal transition. This conserved Draper–ATG3 axis couples phagocytic signaling to autophagy activation, providing mechanistic insight into programmed tissue remodeling in insects.

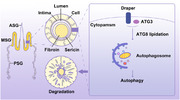

## 1 Introduction

Autophagy transports cytoplasmic materials to lysosomes for degradation in eukaryotic cells,^[^ ^1^ ^]^ aiding cell survival under stress conditions, including nutrient deprivation, and influencing cell death during development and pathogenesis.^[^ ^2^, ^3^, ^4^, ^5^, ^6^ ^]^ Research on degenerating larval tissues in *Drosophila* metamorphosis has revealed the role of autophagy in cell death; autophagy eliminates abnormal or damaged cells and facilitates tissue pattern formation. However, compared with nutrient deprivation‐induced autophagy, the regulation of autophagy during developmental cell death is less well understood.

Insect metamorphosis, one of the earliest processes studied in the context of developmental cell death, involves a series of programmed tissue histolysis and remodeling processes, with autophagy playing a pivotal role in these processes.^[^ ^7^ ^]^ For example, in *Drosophila melanogaster*, the midgut is degraded via autophagy rather than apoptosis.^[^ ^8^ ^]^ Increased autophagosome formation occurs in dying midguts, and the disruption of autophagy via knockout or knockdown of autophagy‐related protein (ATG) 1 or ATG18 markedly impairs midgut removal.^[^ ^9^ ^]^ In contrast, salivary gland removal involves both autophagy and apoptosis.^[^ ^10^, ^11^ ^]^ Autophagy functions as a mechanism for survival in the midgut of silkworm larvae, whereas apoptosis is the primary process governing the destruction of the epithelium.^[^ ^12^ ^]^ In silkworm silk glands, autophagy precedes apoptosis by 48–72 h and provides the energy required for cell survival during the spinning stage.^[^ ^13^ ^]^ Autophagy‐dependent cell death refers to a form of programmed cell death that requires autophagic machinery for its execution, often in a non‐apoptotic manner.^[^ ^14^, ^15^ ^]^ In contrast, adaptive autophagy represents a cellular survival mechanism, where autophagy is transiently activated in response to stressors such as nutrient deprivation, oxidative stress, or infection, thereby promoting homeostasis and adaptation rather than cell death.^[^ ^16^, ^17^ ^]^ Decapentaplegic, a ligand for bone morphogenetic protein/transforming growth factor β in *Drosophila*, inhibits autophagy‐mediated midgut degradation.^[^ ^18^ ^]^ Ubiquitously transcribed tetratricopeptide repeat on chromosome X, a histone demethylase, controls the expression of genes involved in cell death and self‐digestion during salivary gland degradation.^[^ ^19^ ^]^ Ras‐like protein A triggers autophagy‐dependent cell death upon Notch activation.^[^ ^20^ ^]^ Based on these findings, the role of autophagy as a death or survival process in metamorphosis is considered context‐dependent, although the precise regulatory mechanisms remain elusive.

Draper, an ortholog of cell death abnormal (CED)‐1 in *Caenorhabditis elegans* and multiple epidermal growth factor‐like domains protein 10 (MEGF10) in mammals, primarily functions as a receptor for cell engulfment in *D. melanogaster*, aiding in the phagocytosis of apoptotic cells.^[^ ^21^, ^22^ ^]^ Previous studies have highlighted that multiple elements collaborate with Draper to facilitate the engulfment and processing of apoptotic cells in *Drosophila* phagocytes.^[^ ^23^, ^24^, ^25^ ^]^ Knockout or knockdown of *Draper* in *D. melanogaster* salivary glands leads to salivary gland persistence during metamorphosis.^[^ ^26^ ^]^ Draper may non‐cell autonomously regulate autophagy through *Drosophila* macroglobulin complement‐related protein in neighboring cells during wound healing and cell death.^[^ ^27^ ^]^ However, the mechanism underlying autophagy regulation by Draper remains unclear. *Bombyx mori* is a valuable insect for the economy and a typical model for lepidopteran research. Silkworm silk glands produce silk protein and comprise the anterior silk gland (ASG), middle silk gland (MSG), and posterior silk gland (PSG). The MSG produces sericin, a major silk protein component.^[^ ^28^ ^]^ In the ASG and PSG, autophagy and apoptosis coexist during metamorphosis.^[^ ^13^, ^29^ ^]^ However, the molecular mechanisms governing silk gland degradation during metamorphosis remain incompletely understood.

In this study, we employed an integrative approach to elucidate the physiological functions of Draper in insect metamorphosis. First, we reconstructed the evolutionary trajectory of Draper across insect species and subsequently generated *Draper* mutations in *B. mori* using the CRISPR/Cas9 system. We explored the role of the engulfment receptor Draper in orchestrating autophagy‐mediated degeneration of the silk gland during metamorphosis. Draper deficiency significantly delayed MSG degeneration and impaired autophagy induction during metamorphosis. Quantitative proteomics revealed that the loss of Draper resulted in abnormal accumulation of silk‐associated proteins in mutant silk glands. Co‐immunoprecipitation coupled with liquid chromatography–tandem mass spectrometry (LC‐MS/MS) was further performed to find a cohort of autophagy regulators that interact with Draper. Together with evolutionary conservation and CRISPR‐mediated validation, our data establish Draper as a master regulator of spatiotemporal autophagy and tissue remodeling in insect development.

## 2 Results

### 2.1 Draper Is Highly Conserved Across Insect Species

**Figure 1:**
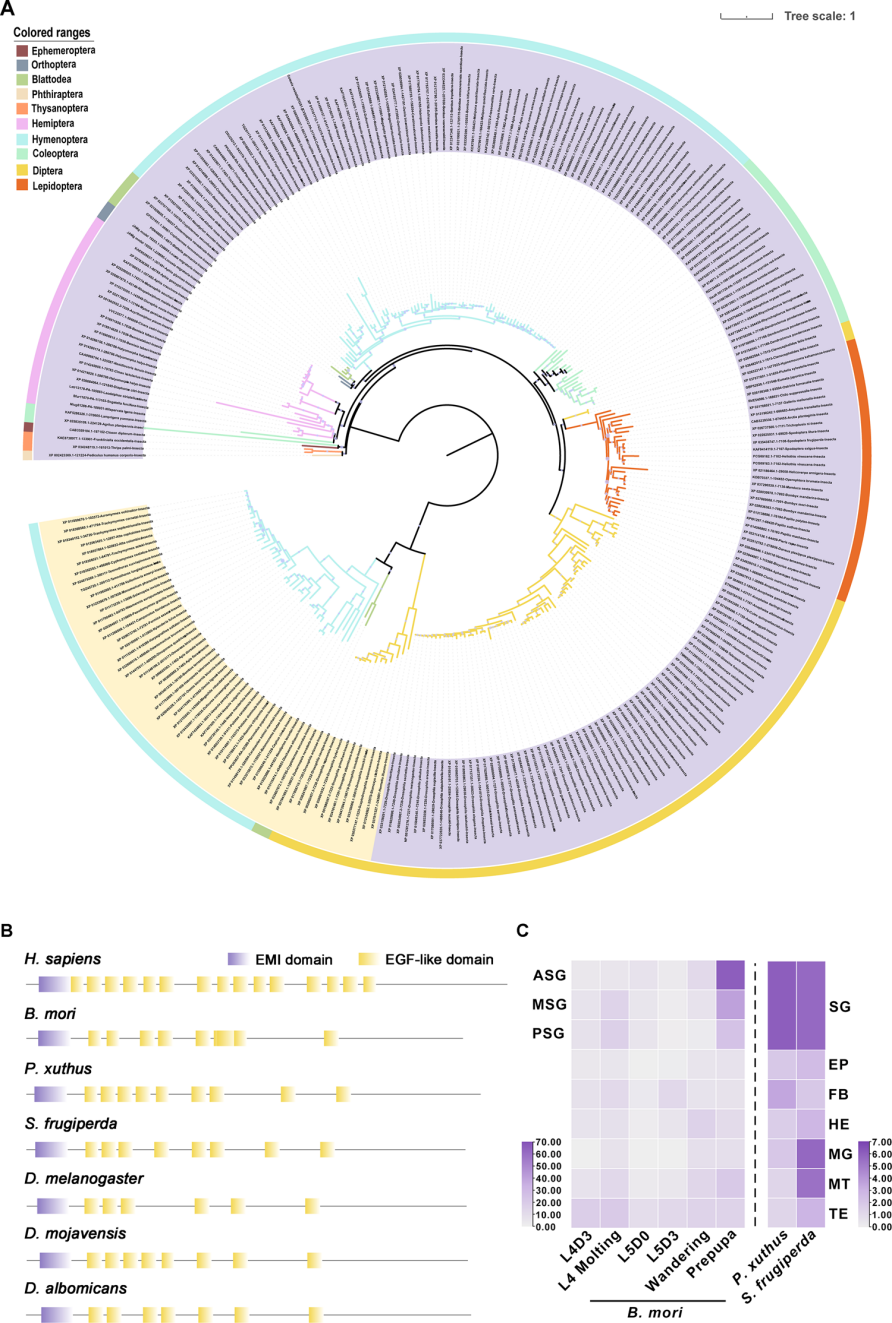
Phylogenetic analysis and tissue expression of *Draper*. A) Maximum likelihood phylogenetic tree of Draper proteins in 202 insect species. The phylogenetic tree was constructed with 277 Draper homologous proteins. Purple and yellow color ranges represent the two branches of Draper proteins in insects. Different colors of the outside circles indicate different insect orders. The purple circles on the branch nodes represent bootstrap values. The smaller the circle, the larger the bootstrap value and the higher the credibility. The credibility of all branches is above 90%. B) Conserved domains of Draper proteins in seven species. The purple rectangle and yellow rectangle represent the EMI domain and EGF‐like domain, respectively. Grey lines represent the amino acid sequence lengths of the corresponding proteins. C) *Draper* expression patterns in *B. mori*, *P. xuthus*, and *S. frugiperda*. Expression data from SilkDB 3.0 (https://silkdb.bioinfotoolkits.net/main/species‐info/‐1) were searched to identify the expression profiles of *Draper* in 54 samples throughout the silkworm across tissues and developmental stages. RNA‐seq data of *P. xuthus* and *S. frugiperda* on the 3rd day of fifth instar larvae were collected to identify the expression profiles of *Draper* in the silk gland (SG), epidermis (EP), fat body (FB), head (HE), midgut (MG), Malpighia tubule (MT), and testis (TE).

Using the *B. mori* Draper sequence, we identified 277 Draper homologs within 202 insect proteomes. Of these, 135 insect species possessed a single‐copy Draper gene, while 68 species harbored multi‐copy genes (Figure S1, Supporting Information). We constructed a maximum‐likelihood phylogeny using the 277 Draper protein sequences (**Figure** **1**A). This phylogeny revealed two distinct Draper clades. Homologs clustered largely by insect order, indicating that Draper is an evolutionarily ancient and ubiquitous insect gene. Notably, multi‐copy *Draper* genes from specific orders were distantly related and formed separate clusters. These findings suggest that while the ancestral *Draper* gene underwent divergence in certain insect lineages, *Draper* is conserved across most insect species.

Draper, a nimrod A‐like protein, contains an EMI domain at the N‐terminal region and one NIM motif as well as a variable number of epidermal growth factor (EGF)‐like domains,^[^ ^30^ ^]^ which are essential for the protein's function.^[^ ^31^ ^]^ Sequence analysis of Draper homologous proteins in three dipteran species and three lepidopteran species, as well as the MEGF10 protein in *Homo sapiens* showed conservation of the EMI domain and EGF‐like domains across Drapers (Figure 1B). The conserved domains of dipteran and lepidopteran Draper proteins were similarly located on their respective sequences with high sequence similarity and conserved sequence composition (Figure S2A,B, Supporting Information), which were important for the maintenance of Draper's functional activity. Overall, these results suggest the functional conservation of Draper in Diptera and Lepidoptera.

Data from SilkBD 3.0 were used to profile *Draper* transcript levels across tissues and stages in *B. mori*. The Draper expression was highest in both the ASG and MSG, peaking at the prepupal stage just before the larval–pupal transition (Figure 1C). We then analyzed published RNA‐seq data from two additional lepidopterans: *Papilio xuthus* (Lepidoptera: Papilionidae) and *Spodoptera frugiperda* (Lepidoptera: Noctuidae). In both species, Draper exhibited high expression in silk glands, with detectable expression also in malpighian tubules and midgut during late‐instar larvae (Figure 1C). This conserved expression pattern across three lepidopteran species suggests Draper's role in silk gland physiology is conserved.

### 2.2 Draper Plays a Crucial Role in MSG Degradation and Silk Properties

**Figure 2:**
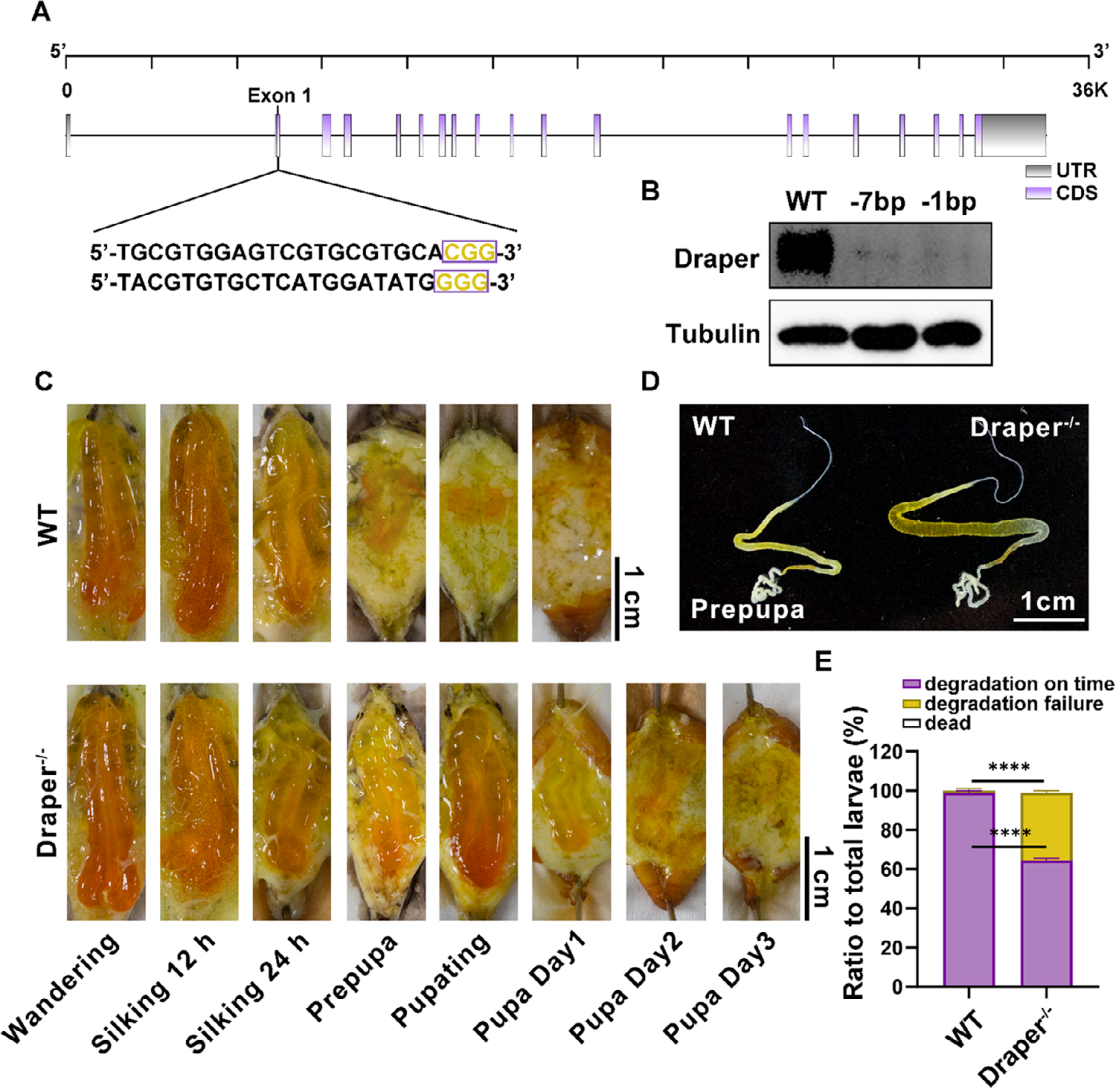
Knockout of *Draper* and phenotype observation in *B. mori*. A) Schematic illustration of *Draper* and the sgRNA targets. Grey and purple squares represent the untranslated regions (UTR) and coding sequences (CDS), respectively. Two sgRNA targeting sites were found on the first exon. The sgRNA sequences and corresponding PAM sequences are shown in black and yellow, respectively. B) Immunoblot validation of two *Draper* mutants. C) Morphology of wild‐type and *Draper* mutant silk glands. Upper and lower panels show the silk glands from wild‐type individuals and *Draper* mutants, respectively, at each stage. Scale bar: 1 cm. D) Silk glands phenotypes observed at the prepupal stage. E) Ratio of silk glands with incomplete degradation at the prepupal stage. Scale bar: 1 cm. Thirty larvae per group in each experiment were randomly selected and dissected at the prepupal stage. Individuals with degradation failure were counted.

To investigate the physiological roles of Draper, we generated *Draper* mutants in *B. mori* using the CRISPR/Cas9 system. Injection of two *Draper*‐targeting single guide RNAs (sgRNAs) into early embryos resulted in two different chromosomal deletions (−7 bp and −1 bp deletions) (**Figure** **2**A; Figure S3 and Table S1, Supporting Information). Dissection of silk gland tissue from two *Draper* mutant lines revealed complete disappearance of Draper protein levels (Figure 2B). Further analysis focused on mutants with the −7 bp deletion due to phenotypic resemblance between two homozygous individuals. *Draper* mutants exhibited significantly delayed degradation of silk glands compared with wild‐type individuals, with 34% (*n* = 30) of *Draper* mutant silk glands remaining intact at the prepupal stage and degrading completely by 3 days after puparium formation (Figure 2C,E). In contrast, most wild‐type silk glands degraded completely on the first day of the pupal stage. Notably, the most significant delay occurred in MSG degradation (Figure 2D). These findings implicate Draper in silk gland degradation.

**Figure 3:**
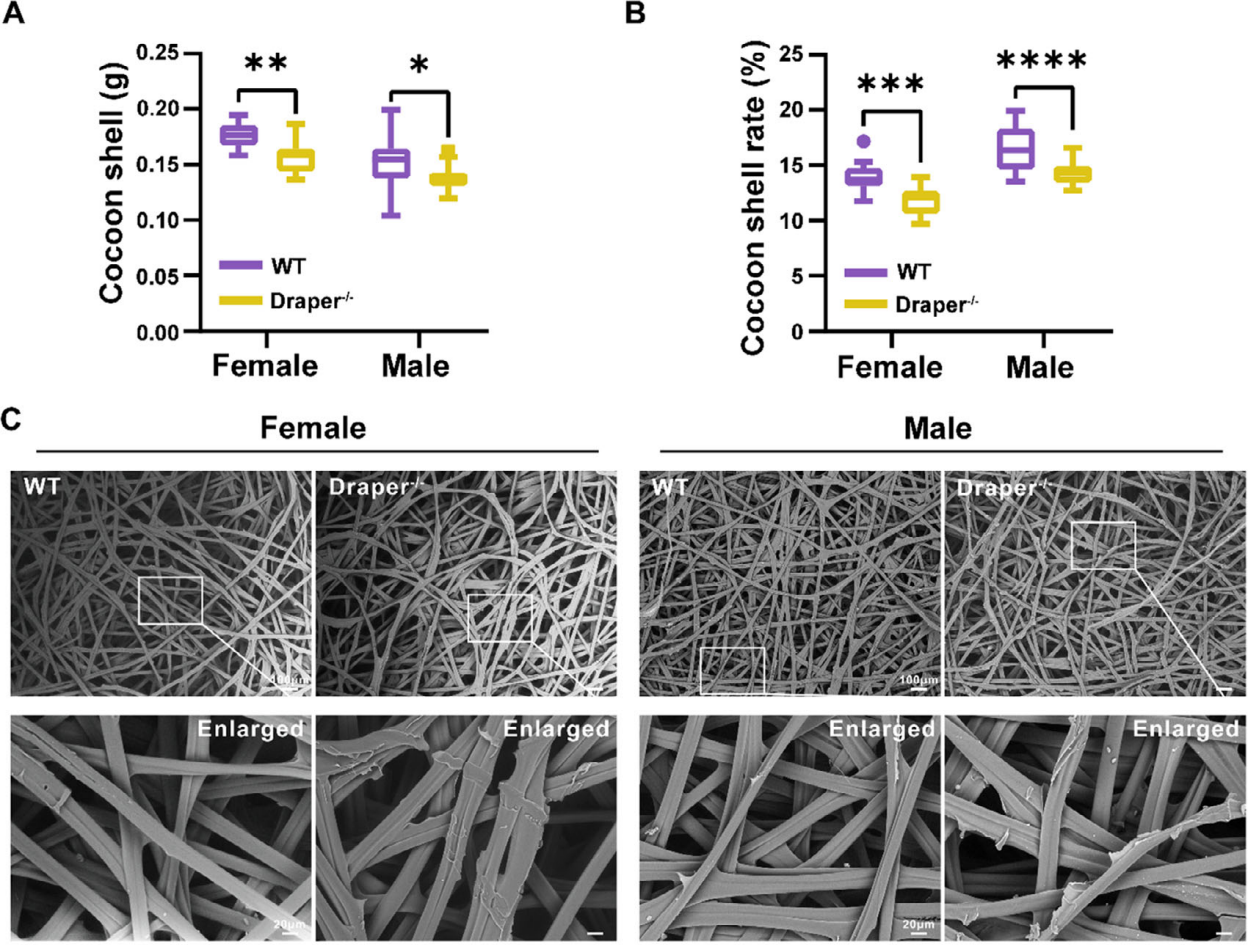
Properties of cocoons and silk fibers. A,B) The weight and rate of cocoon shells for the wild‐type and *Draper* mutant groups. Thirty similarly shaped cocoon in each experiment were randomly selected from a group. The cocoon shell rate was the ratio of cocoon shell weight to cocoon weight. C) Micrographs of representative SEM images demonstrating silk fibers on the surface of cocoons from female and male silkworms. Original scale bar: 100 µm. Enlarged scale bar: 20 µm.

Further analysis of cocoon shell weights and rates revealed significantly lower values in female and male *Draper* mutants than in their wild‐type counterparts (**Figure** **3**A,B). In addition, scanning electron microscopy (SEM) revealed the presence of irregular nodules in the sericin structure of *Draper* mutant silk fibers (Figure 3C). These observations suggest that disruption of *Draper* affects silk qualities and structures.

### 2.3 Draper Promotes Autophagy in the MSG During Larval–Pupal Metamorphosis

**Figure 4:**
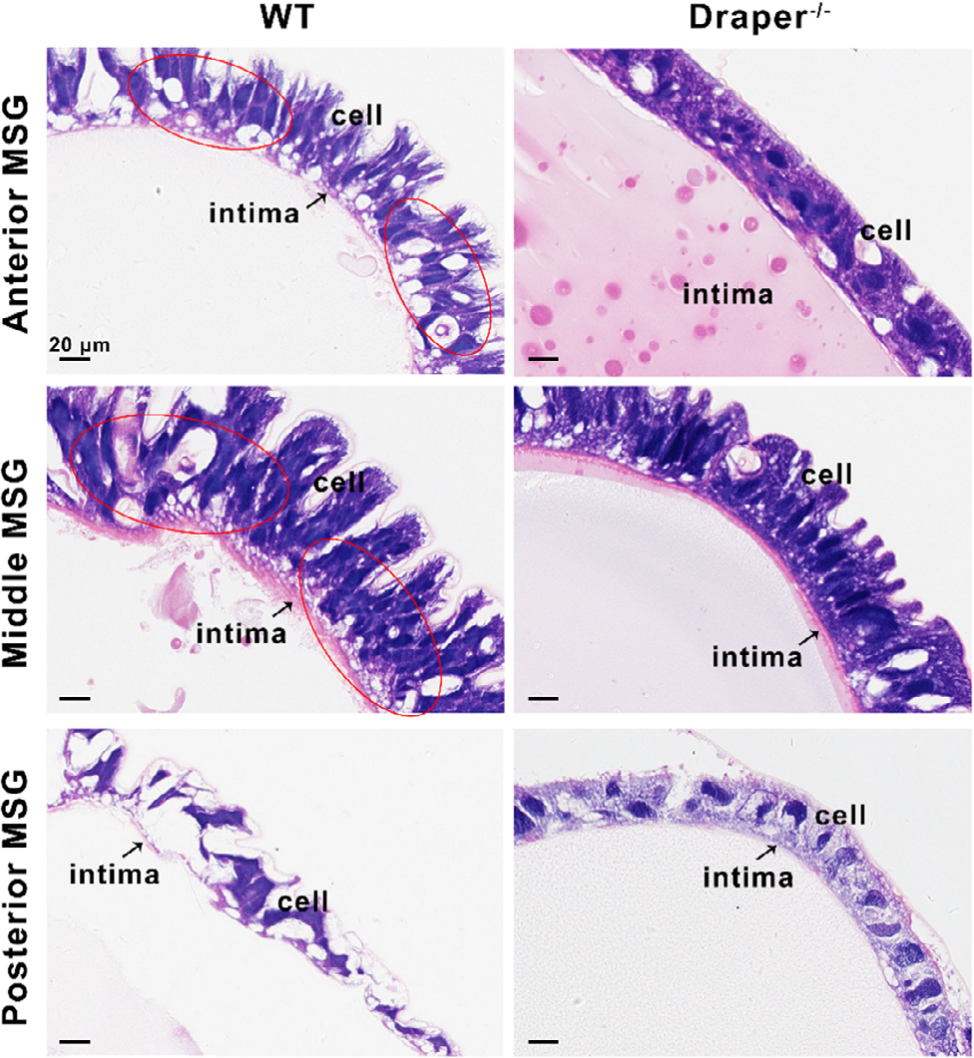
Histomorphological images of three regions of MSG at the prepupal stage. HE staining of the cross‐section shows the tissue histology of three regions of MSG at the prepupal stage, including the anterior MSG, middle MSG, and posterior MSG. Red circles indicate vacuolar structures. Black arrows indicate the intima of MSG. Scale bar: 20 µm.

We observed that the most pronounced delay in gland degradation in *Draper* mutants occurred in the middle silk gland (Figure 2D). We therefore examined the morphology of the anterior, middle, and posterior regions of the middle silk gland at the prepupal stage using hematoxylin and eosin (HE) staining. In wild‐type individuals, epithelial cells in the anterior and middle MSG showed pronounced shrinkage and cytoplasmic vacuolization, accompanied by pallor of the tissue. In the posterior region, epithelial cells frequently detached from the basement membrane, resulting in visible intercellular gaps (**Figure** **4**). These morphological changes are indicative of epithelial remodeling and tissue degeneration during the larval–pupal transition. By contrast, *Draper* mutants exhibited markedly reduced vacuolization and maintained more intact epithelial architecture across all MSG regions, suggesting a significant delay in tissue‐degenerative remodeling in the absence of Draper (Figure 4).

**Figure 5:**
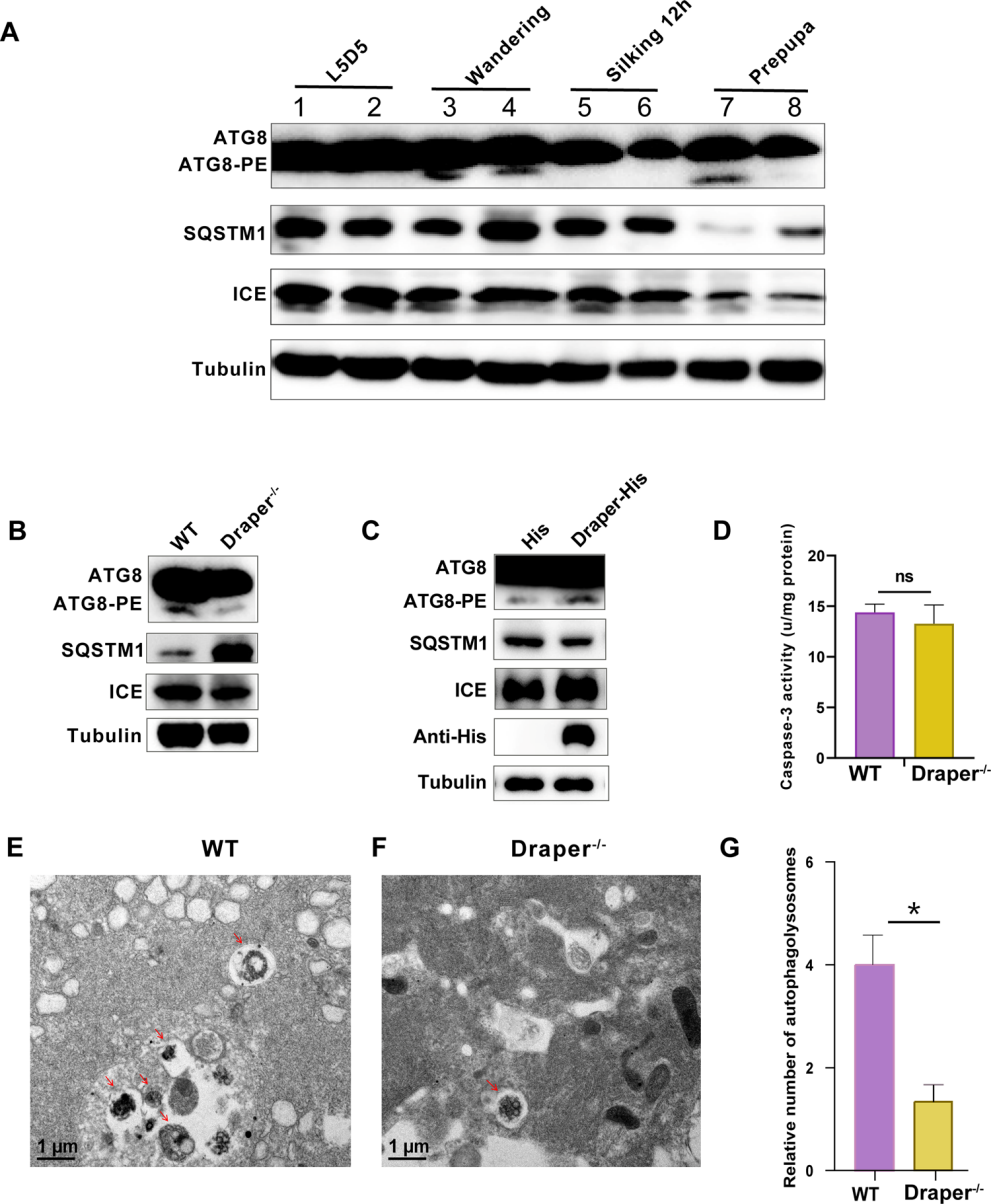
Draper promotes autophagy during the degeneration of MSG. A) ATG8, SQSTM1, and ICE protein levels of MSG during the spinning stage. B) ATG8, SQSTM1, and ICE protein levels of MSG at prepupal stage. C) Protein levels of ATG8, SQSTM1, ICE, and anti‐His after overexpressing Draper in BmN cells. D) Caspase‐3 activity was detected in the wild‐type and *Draper* mutant MSGs. E,F) TEM images of autolysosomes in MSG at the prepupal stage. Autolysosomes are marked by red arrows. Scale bar:1 µm. G) Quantification of autolysosomes in wild‐type and *Draper* mutant MSGs. Autolysosomes were counted from three TEM images of the middle silk gland per group. Data are shown as mean ± SEM. *p* < 0.05 (^*^).

The reduced cell death features in *Draper* mutants suggested that Draper affected autophagy in the MSG. ATG8, the first molecule found to localize to autophagosome intermediate structures, binds to PE to form the ATG8–PE adduct.^[^ ^32^ ^]^ Compared with that in the late larval stage, ATG8–PE formation significantly increased in both wild‐type and *Draper* mutant individuals from the wandering stage, indicating autophagy activation (**Figure** **5**A). In addition, the autophagic receptor sequestosome 1 (SQSTM1) protein accumulated in the silk gland of *Draper* mutants from the wandering stage until the prepupal stage, indicating that autophagic activity in *Draper* mutants was inhibited during the spinning stage (Figure 5A). Furthermore, ATG8–PE formation was significantly reduced in *Draper* mutants compared with that in wild‐type individuals at the prepupal stage; this reduction was accompanied by a significant increase in SQSTM1 levels (Figure 5B). These results demonstrated that the absence of *Draper* significantly inhibits MSG autophagy at the prepupal stage. Transmission electron microscopy (TEM) revealed the presence of autophagosomes and autolysosomes in the MSG tissues of wild‐type individuals at the prepupal stage, whereas *Draper* mutants exhibited fewer autophagy structures (Figure 5E–G). These findings suggest that Draper promotes autophagy in the silk gland during the prepupal phase.

To confirm the role of Draper in autophagy, we examined autophagic activity after overexpressing Draper in BmN cells. Decreased SQSTM1 protein levels and increased ATG8–PE formation in BmN cells indicated the positive impact of Draper on autophagy (Figure 5C). Interleukin‐1β converting enzyme (ICE), a cysteine protease belonging to the caspase family in silkworm and containing specific aspartic acid, is an executive caspase that promotes cell apoptosis.^[^ ^33^ ^]^ No significant difference in ICE protein levels was detected between wild‐type individuals and *Draper* mutants during the spinning stage (Figure 5A,B). Similarly, overexpression of Draper in BmN cells did not result in any significant alteration in ICE protein levels (Figure 5C). Moreover, Caspase‐3 activity remained comparable between wild‐type individuals and *Draper* mutants at the prepupal stage (Figure 5D). These collective findings demonstrate that Draper has a negligible impact on apoptosis in degenerating MSG cells. Taken together, our results provide compelling evidence that Draper plays a crucial role in mediating autophagy during MSG degradation.

### 2.4 Draper Affects the Ubiquitin System and Silk Protein Metabolism in the MSG

To understand the function of Draper in the MSG at the prepupal stage, we used 4D label‐free quantitative proteomics on wild‐type and *Draper* mutant groups (Figures S4 and S5, Supporting Information). 2124 proteins were identified from six samples, comprising 1936 identical proteins, 156 wild‐type‐specific proteins, and 32 mutant‐specific proteins (Figure S6A, Supporting Information). In addition, 746 differentially expressed proteins (DEPs) were identified in the MSG of *Draper* mutants compared with wild‐type individuals, meeting screening criteria of fold change > 1.2 or < 0.83 and *p* < 0.05 (Figures S4C and S5 and Table S2, Supporting Information). Among these DEPs, 263 and 483 were upregulated and downregulated, respectively (Figure S6C, Supporting Information).

To elucidate the functional importance of DEPs, we conducted Gene Ontology (GO) enrichment analysis. We manually categorized GO terms associated with primary functional groups to examine classification characteristics in enriched terms. The 263 upregulated DEPs were significantly enriched for 39 GO terms: 12 terms were related to metabolic process, primarily involving nucleotide metabolism, carbohydrate metabolism, lipid metabolism, and nitrogen compound metabolism; 12 terms were related to biosynthetic process, mainly involving nucleotide biosynthesis and nitrogen compound biosynthesis; and 6 terms were involved in responses, predominantly concerning stimulus and immunity responses (Figure S6B and Table S3, Supporting Information). The 483 downregulated DEPs were significantly enriched for 49 GO terms: 8 terms were associated with metabolism and biosynthesis, mainly covering nucleotide metabolism and lipid biosynthesis; 7 terms were related to biomolecule transport and signal transduction, primarily involving nucleotide transport; and 3 terms were involved in responses, mainly associated with stimulus (Figure S6E and Table S4, Supporting Information). These results suggested that the loss of *Draper* affects both the metabolism and synthesis of nucleotides, nitrogen compounds, and lipids as well as responses to stimuli and immunity. Ubiquitination is a reversible post‐translational modification that regulates diverse cellular processes through signaling pathways and maintains cellular homeostasis.^[^ ^34^, ^35^, ^36^, ^37^ ^]^ Notably, ubiquitin‐associated processes were exclusively enriched in the GO analysis of downregulated DEPs. Key ubiquitination machinery components—including ubiquitin‐activating enzyme (E1), ubiquitin‐conjugating enzyme (E2), and ubiquitin ligase (E3)—showed significant downregulation in *Draper* mutant silk glands (Figure S6D, Supporting Information), suggesting systemic suppression of the ubiquitin pathway.

We then conducted Kyoto Encyclopedia of Genes and Genomes (KEGG) enrichment analysis to investigate the signaling pathways influenced by Draper. In total, 23 and 6 KEGG pathways were significantly enriched for the 263 upregulated DEPs and 483 downregulated DEPs, respectively (Figure S7A, Supporting Information). Upregulated DEPs were significantly enriched in metabolic pathways, lysosomes, phagosomes, and mitophagy. The metabolic pathways included amino acid and glucose metabolism. Glucose metabolism is used to generate energy in the form of adenosine triphosphate.^[^ ^38^ ^]^ The mobilization of glycogen is known to provide energy sources required for silk gland metamorphosis.^[^ ^13^ ^]^ The KEGG analysis suggested that loss of Draper affected both protein and energy metabolism.

We identified 49 DEPs related to silk proteins in the MSG, with sericin 1, sericin 3, fibroin heavy chain precursor, and fibroin light chain precursor showing upregulation in *Draper* mutants (Figure S7B, Supporting Information). In addition, protease inhibitors, including BCP inhibitor precursor and serine protease inhibitor swm‐1, were also significantly upregulated in these mutants (Figure S7B, Supporting Information). Accumulated silk proteins likely contribute to MSG integrity in *Draper* mutants. SQSTM1, an autophagic substrate, links and recruits cytosolic cargo material to autophagic membranes.^[^ ^39^ ^]^ The protein level of SQSTM1 was significantly upregulated in the *Draper* mutants (Figure S7B, Supporting Information). These results suggest that loss of Draper caused silk protein accumulation, thereby delaying MSG degradation.

### 2.5 Draper Interacts with ATG3 to Positively Regulate Autophagy

**Figure 6:**
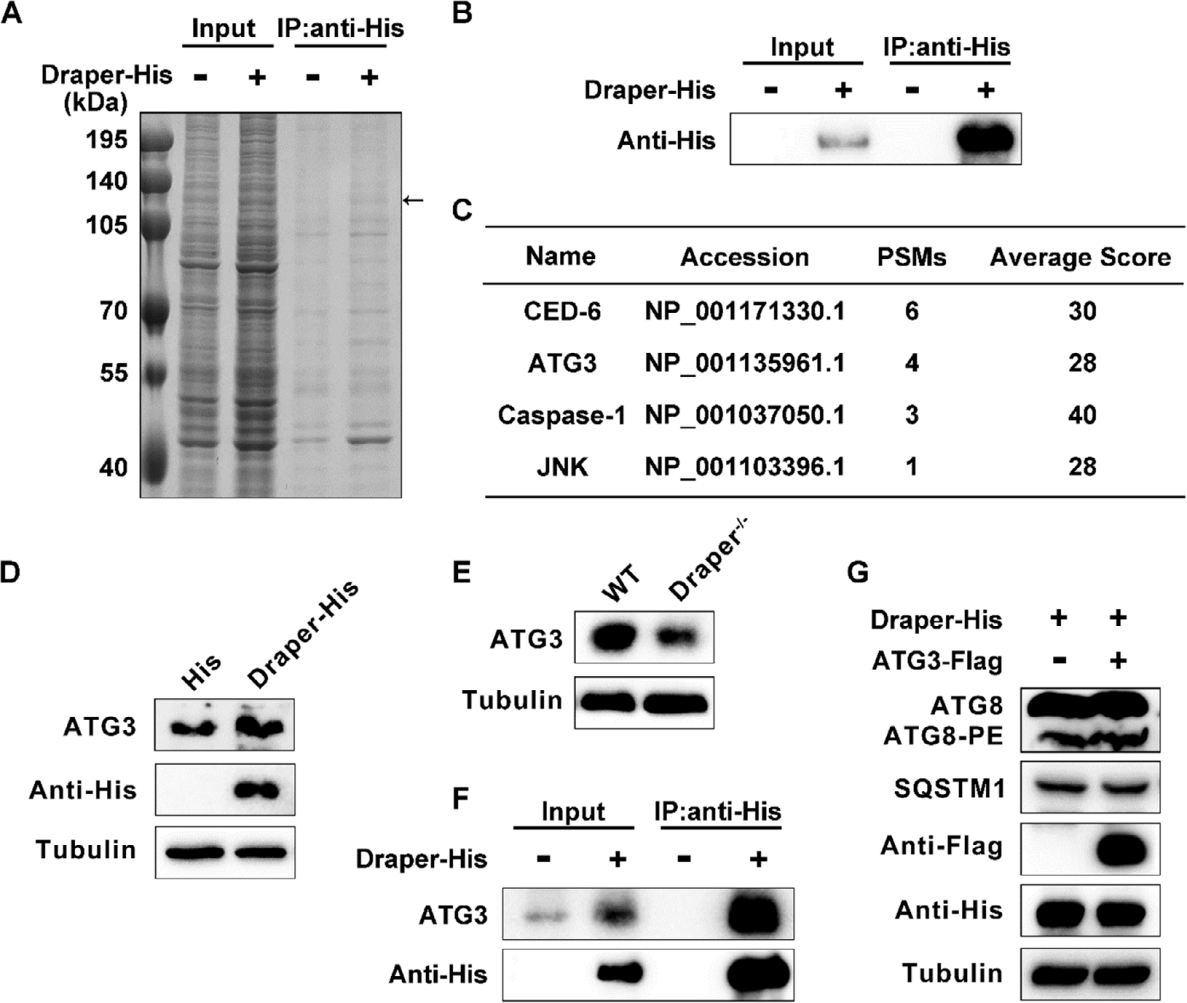
Identification of Draper‐interacting proteins. A) Sodium dodecyl sulfate‐polyacrylamide gel electrophoresis gel image of samples following exogenous Draper immunoprecipitation. The black arrow indicates the Draper protein band. B) Western blotting analysis of Draper–His expression post‐immunoprecipitation. C) Detection of interacting proteins in full gels via LC–MS/MS. PSMs: peptide spectrum matches. D) Protein levels of ATG3 and Draper after Draper overexpression in BmN cells. E) ATG3 protein level in the MSG at the prepupal stage. F) Co‐IP analysis of Draper in BmN cells. Immunoprecipitation was performed using anti‐His magnetic beads on lysates of BmN cells expressing Draper, followed by immunoblotting with ATG3 antibody. G) Protein levels of ATG8, SQSTM1, ATG3, and Draper following expression of Draper and coexpression of Draper and ATG3 in BmN cells.

To explore whether the function of Draper in autophagy can be influenced by its interacting proteins, we isolated total proteins from BmN cells expressing Draper–His and performed an immunoprecipitation assay using anti‐His magnetic beads (**Figure** **6**A). Immunoprecipitated Draper was confirmed using an anti‐His antibody, and the full gel band after immunoprecipitation was analyzed using LC–MS/MS (Figure 6B).

We identified 388 proteins as putative Draper interactors (Tables S5 and S6, Supporting Information), including the previously reported partners CED‐6, Caspase‐1, and JNK (Figure 6C). Among these were autophagy‐related proteins not previously linked to Draper function, such as ATG3. This E2‐like enzyme mediates ATG8 conjugation to autophagosome membranes, an essential step in autophagy.^[^ ^40^ ^]^ Western blot analysis showed that Draper overexpression upregulated ATG3 protein levels in *B. mori* BmN cells, whereas Draper deficiency led to reduced ATG3 levels (Figure 6D,E).

To investigate a potential physical interaction, we performed coimmunoprecipitation (Co‐IP) using anti‐His magnetic beads followed by immunoblotting with an anti‐ATG3 antibody. Draper and ATG3 were coimmunoprecipitated, supporting their interaction in vivo (Figure 6F). However, coexpression of Draper and ATG3 in BmN cells did not significantly enhance ATG8–PE formation or SQSTM1 degradation, suggesting that Draper may regulate autophagy through modulating ATG3 stability or localization (Figure 6G). Taken together, these results indicate that Draper may interact with ATG3 and positively modulate autophagy.

## 3 Discussion

In insects undergoing complete metamorphosis, autophagy is pivotal for remodeling larval tissues and organs.^[^ ^41^, ^42^, ^43^ ^]^ However, how autophagy mechanistically regulates cell death and tissue remodeling remains unclear. Here, we demonstrate that loss of Draper causes a marked delay in middle silk gland degradation during metamorphosis. Ultrastructural analysis revealed reduced numbers of autophagosomes and autolysosomes in *Draper* mutant MSG compared with wild‐type. Furthermore, Draper disruption impeded ATG8–PE conjugation and elevated SQSTM1 expression in *Draper* mutants at the prepupal stage. Critically, we identified an interaction between Draper and ATG3, which positively regulates autophagy. These findings elucidate how developmental signals mechanistically link to core autophagy machinery to ensure precise tissue remodeling.

Phylogenetic analysis indicates evolutionary conservation of Draper across insect species. Consistently, *Draper* exhibits high expression in silk glands of three lepidopterans, suggesting functional conservation in this tissue. Silk glands degenerate completely during metamorphosis,^[^ ^44^ ^]^ concurrent with silk spinning.^[^ ^45^ ^]^ In *B. mori*, Draper loss markedly delays middle silk gland clearance; mutants retain intact MSGs at spinning completion, demonstrating its necessity for MSG degradation. Consequently, cocoon weight and spinning rate decrease. These defects may arise because impaired autophagy in Draper‐deficient glands permits accumulation of damaged secretory organelles and misfolded silk proteins, compromising fiber assembly.^[^ ^26^ ^]^ Proteomic data confirms silk‐related protein accumulation in the mutants, accounting for persistent MSG integrity.^[^ ^46^ ^]^ Collectively, these findings establish Draper's essential role in MSG degeneration during silkworm metamorphosis. However, the molecular mechanistic basis for Draper's pleiotropic effects requires further investigation.

Autophagy and apoptosis coexist in various tissues in both *Drosophila* and lepidopterans during metamorphosis.^[^ ^47^ ^]^ Although research on dying larval tissues in *Drosophila *metamorphosis has implicated autophagy in cellular demise, the precise mechanism remains incompletely understood. Our findings reveal that autophagy was activated in the MSG during the spinning stage, consistent with previous reports regarding the PSG.^[^ ^13^ ^]^ The absence of Draper significantly reduced the number of autophagic vesicles and autophagosomes in the MSG. Notably, ATG8 lipidation and SQSTM1 degradation increased in *Draper* mutants at the prepupal stage, with proteomics analysis also showing that SQSTM1 levels increased in *Draper* mutants. Conversely, apoptotic features didn't show significant changes in *Draper* mutants compared to wild‐type at the prepupal stage. This indicates minimal impact of Draper loss on caspase activity, consistent with reports in *D. melanogaster* salivary glands.^[^ ^48^ ^]^ Apoptosis typically activates after autophagy in lepidopteran metamorphosis.^[^ ^49^ ^]^ Although silk gland degradation was impaired in *Draper* mutants, caspases persisted, suggesting Draper acts downstream of or parallel to caspases in silk gland cell death. These findings imply cooperative functions for caspases and autophagy during gland degradation, accounting for eventual MSG disappearance in mutants. Collectively, Draper‐dependent signaling emerges as essential for effective autophagy and subsequent MSG degradation. For example, Draper and CED‐6 show a genetic interaction in larval axon glial engulfment during *Drosophila* metamorphosis, a process crucial for apoptotic cell clearance in *C. elegans*.^[^ ^50^ ^]^ In addition, nurse cell death induced by Draper relies on Caspase‐1, suggesting a comparable apoptotic pathway to starvation‐induced cell death.^[^ ^51^ ^]^ Moreover, Draper triggers *Drosophila* JNK signaling in ensheathing glia and astrocytes, playing a crucial role in efficient apoptotic neuron removal.^[^ ^52^ ^]^ Although we establish the Draper‐ATG3 axis as critical for autophagy initiation, the complete degradation of silk glands likely requires coordinated crosstalk between autophagy and other cell death pathways.

Autophagy is associated with various cellular pathways through interactions with key genetic components.^[^ ^53^ ^]^ Interacting partners may regulate the role of Draper in autophagy. Although previous studies have reported the interaction of Draper with several adapter proteins,^[^ ^50^, ^51^, ^52^, ^54^ ^]^ the mechanism underlying Draper's transduction of autophagy component activation into the autophagy process remains elusive. We herein demonstrated that Draper interacts with ATG3 to positively regulate autophagy. ATG3 is a key player in autophagy, participating in ATG8–PE conjugation, with homologs found across many eukaryotic organisms.^[^ ^55^ ^]^ ATG3 induces autophagy by increasing ATG8 lipidation.^[^ ^56^ ^]^ Increased ATG3 expression contributes to autophagy triggered by the separation of intestinal epithelial cells from the extracellular matrix.^[^ ^57^ ^]^ Interacting partners can regulate ATG3 function in autophagy. For example, viral FLICE‐inhibitory protein, a viral counterpart of cellular FLICE‐inhibitory protein produced by human herpesvirus 8, inhibits cellular antiviral autophagy by interacting with ATG3.^[^ ^58^ ^]^ In *Nicotiana benthamiana* plants, cytosolic glyceraldehyde‐3‐phosphate dehydrogenases interact with ATG3, thereby inhibiting autophagy.^[^ ^59^ ^]^ In the present study, LC–MS/MS analysis and Co‐IP assays revealed the interaction of Draper with ATG3. In addition, Draper overexpression elevated ATG8–PE and SQSTM1 protein levels, whereas Draper and ATG3 coexpression did not lead to an increase in these levels, suggesting that Draper interacts with ATG3 to promote ATG3‐induced autophagy. While the Draper‐ATG3 interaction is established, elucidating how this interaction modulates ATG3's enzymatic activity or spatial dynamics during autophagosome biogenesis will require detailed future structural and live imaging analyses.

### 3.1 Conclusion

**Figure 7:**
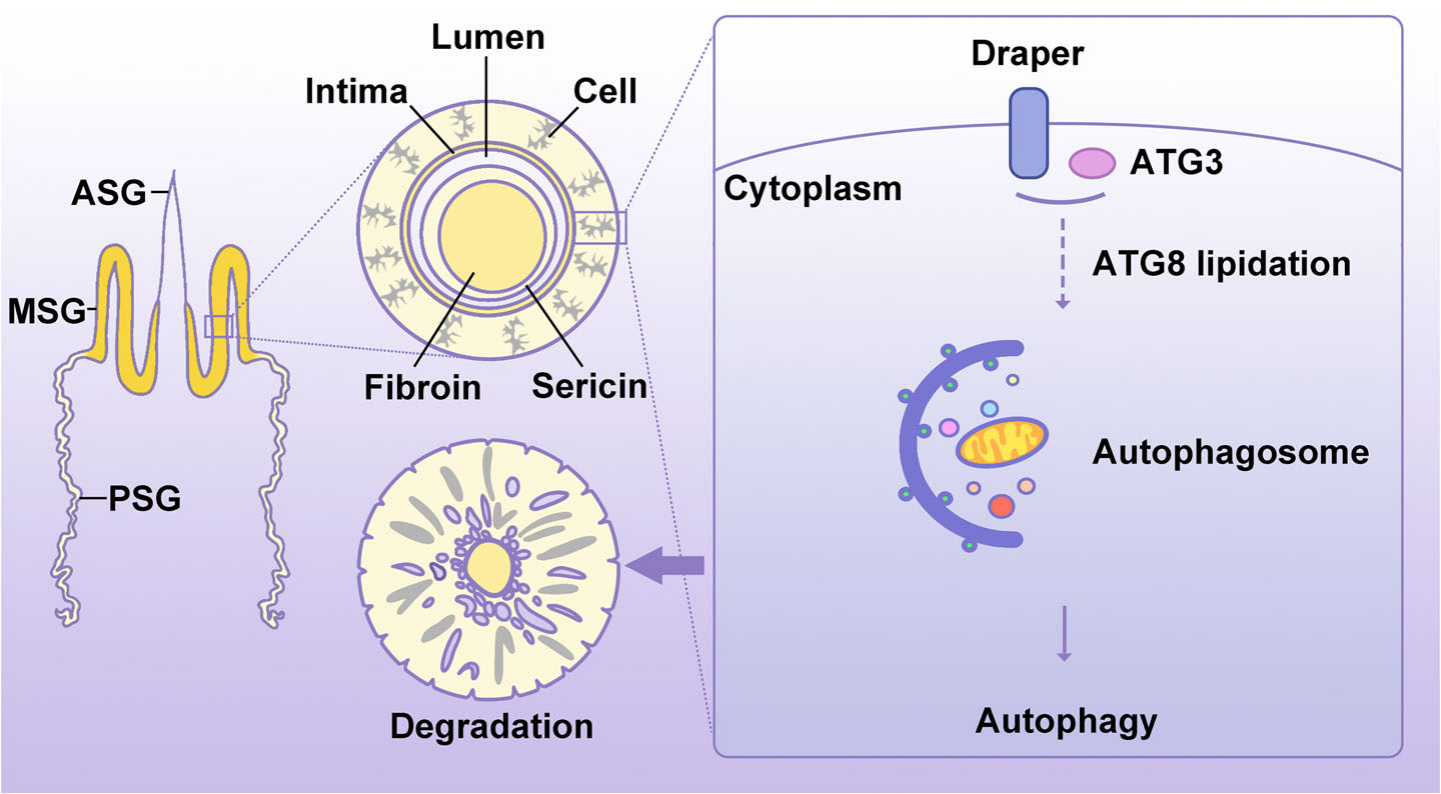
Schematic representation of Draper in regulating autophagy and subsequent degradation of MSG. ATG3 promotes autophagy by inducing ATG8 lipidation. Draper interacts with ATG3 to positively regulate autophagy and then promote the degradation of MSG.

Using an integrative approach combining evolutionary trajectory analysis, CRISPR‐Cas9‐mediated genome editing, proteomic profiling, and functional validation, we uncover that Draper mediates the spatiotemporal control of autophagy during developmental cell death in the silk gland. We demonstrate that Draper recruits ATG3 to orchestrate autophagosome formation, a process essential for silkgland degradation during the larval‐pupal transition (**Figure** **7**). Collectively, these findings advance our understanding of autophagy's role in the complex regulation of insect tissue removal and remodeling. The discovery of Draper as an evolutionarily conserved regulator challenges the current paradigm of autophagy initiation and provides a unifying framework linking developmental timing, phagocyte signaling, and metabolic clearance in metamorphosis.

## 4 Experimental Section

### Insect Materials and Cell Lines

Larvae of *S. frugiperda* were fed with an artificial diet, larvae of *P. xuthu* were reared on fresh Citrus leaves, and larvae of *B. mori* were fed with fresh mulberry leaves. All stages were kept under standard conditions of 26 ± 1 °C, with a relative humidity of 70 ± 5% and a 14:10 h (L: D) photoperiod. BmN cells were cultured in Sf‐900 II SFM medium (Thermo Fisher Scientific) supplemented with 3% fetal bovine serum at 27 °C as previously described.^[^ ^60^ ^]^

### Phylogenetic Analysis and Sequence Analysis of Draper Proteins

We performed a sequence alignment with a database of 202 insect proteins to determine the distribution and evolutionary connections of Draper in insect species as previously described.^[^ ^61^ ^]^ The amino acid sequence from *B. mori* Draper was utilized as a query and retrieved 277 homologous sequences of 202 insects via the BLASTP program. We then manually aligned and removed incomplete and redundant sequences to obtain the Draper homologous proteins in insects. To compare differences in *Draper* gene copy number across insect orders, the gene numbers in each insect species were counted and visualized using iTOL (http://itol.embl.de). A phylogenetic tree was built using the amino acid sequences of 277 Draper homologous proteins as previously described.^[^ ^62^ ^]^ The phylogenetic tree was marked and modified by using iTOL and Adobe Photoshop CC 2019 (v20.0.1). Accession numbers for all sequences are shown in Table S7 (Supporting Information).

To analyze protein sequences of Drapers, the conserved domains of the MEGF10 protein (XP_01 686 5476.1) and some identified insect Draper proteins were searched in the UniProt online database (https://www.uniprot.org) and visualized with iTOL.

### Tissue Expression Analysis of Draper

On the 3rd day of the fifth larval stage, we dissected different tissues to analyze the transcriptional profiles of the *Draper* gene in various tissues (including silk gland, epidermis, fat body, head, midgut, Malpighian tubule, and testis) from *P. xuthu* and *S. frugiperda*. Following the guidelines provided by the manufacturer, the samples were mixed with TRIzol reagent (Takara) for homogenization. Multikan GO (Thermo Scientific) was used to measure the RNA concentration. Each group underwent three biological replications. We performed transcriptome sequencing as previously described.^[^ ^63^ ^]^ RNA‐Seq data were utilized to create a heatmap displaying the expression level of the *Draper* gene using TBtools, followed by graph plotting in Adobe Photoshop CC 2019.

### Establishment of *Draper* mutants

*Draper* mutants were established using the CRISPR/Cas9 system as previously described.^[^ ^64^ ^]^ SgRNAs were synthesized in vitro using the HiScribe T7 Quick High Yield RNA Synthesis Kit (New England Biolabs) following the manufacturer's protocol, and purified using ethanol precipitation.^[^ ^65^ ^]^ The list of designed sgRNAs sequences was listed in Table S1 (Supporting Information). Custom sgRNAs were synthesized and transcribed as previously described.^[^ ^66^ ^]^ The injection mixture consisted of 500 ng sgRNA, 0.3 µL Cas9 protein, and nuclease‐free water to a total volume of 5 µL. Approximately 2 nL of the mixture was microinjected into each fertilized egg within 6 h of oviposition. A total of 500 eggs were injected. Injected eggs were then maintained in an incubator at 26 °C  ±  1 °C with a relative humidity of 60% ± 10% until hatching.

DNA was extracted from the legs of individual G0 moths and subjected to PCR amplification and Sanger sequencing using primers listed in Table S1 (Supporting Information). Mosaic sequence peaks indicated successful mutation events.^[^ ^67^ ^]^ Putative G0 mutants were individually crossed with wild‐type moths to produce G1 offspring. Sequencing of G1 individuals was used to confirm germline transmission of mutations. G1 heterozygotes with the same indel mutation were intercrossed to generate G2, and G2 homozygotes were screened and confirmed by Sanger sequencing and western blotting. This process was repeated for two independent indel variants, resulting in two separate homozygous mutant lines.^[^ ^66^, ^68^ ^]^ In addition, silk glands were dissected, homogenized, lysed, and analyzed via western blotting using antiserum targeting Draper. Homozygous mutants were identified based on the presence of a single sequence peak and the absence of signal detection in western blotting.

Thirty individuals from either the wild‐type or CRISPR/Cas9‐mediated mutants were randomly chosen from a group and fed with quantitative mulberry leaves to study the growth of silk glands. The silk gland was dissected and isolated to observe morphological characteristics during the spinning stage.

### Measurement of Cocoon Parameters

Ten similarly shaped cocoons from the wild‐type or *Draper* mutants were randomly selected from a group. After carefully cutting open the cocoons, the pupae were separated, and then the weight of the cocoons and cocoon shells was measured. The cocoon shell rate was calculated as the cocoon shell weight divided by the total cocoon weight. The weight parameters were measured in grams using an electronic digital balance.

### SEM Analysis

The fibers were analyzed using the SU8010 field emission SEM (Hitachi, Ibaraki, Japan) as previously described.^[^ ^69^ ^]^

### HE Staining

At the prepupal stage, the MSGs were carefully dissected and separated into three parts: the anterior, middle, and posterior regions of the MSG. Each of the three sections was fixed with 4% paraformaldehyde for 24 h and then embedded in paraffin wax. After being exposed to 10% formaldehyde, the samples were subsequently stained with HE.

### TEM Analysis

TEM analysis was conducted as described previously with some modifications.^[^ ^70^ ^]^ MSGs were fixed overnight at 4 °C in 2.5% glutaraldehyde solution. After fixation, the samples were rinsed three times (15 min each) with 0.1 m phosphate‐buffered saline (PBS, pH 7.0). Post‐fixation was carried out with 1% osmium tetroxide (OsO_4_) for 1–2 h at room temperature, followed by three additional rinses in 0.1 m PBS (15 min each). Dehydration was carried out by exposing the samples to escalating ethanol concentrations (30%, 50%, 70%, 80%) for 15 min each. Afterward, the specimens were dehydrated using acetone solutions of 90%, 95%, and 100% for a duration of 15 min. The specimens were then infiltrated with a mixture of Spurr's resin and acetone at graded ratios, embedded in pure resin, and polymerized at 70 °C overnight. Ultrathin sections were obtained using a LEICA EM UC7 ultramicrotome (Leica, Vienna, Austria), stained sequentially with uranyl acetate and lead citrate, and examined using a Hitachi H‐7650 transmission electron microscope (Hitachi, Ibaraki, Japan).

### Caspase‐3 Activity Assay

Caspase‐3 activity was detected using a Caspase‐3 activity kit (Dalian Meilun Biotechnology Co. Ltd., MA0329). Samples were gathered and rinsed using a PBS solution. The protein was then quantified using a Bradford protein assay kit (Dalian Meilun Biotechnology Co. Ltd., MA0081). The assay for Caspase‐3 activity was conducted according to the guidelines provided by the manufacturer. The activity of Caspase‐3 was adjusted based on the total protein content in the sample. Three separate trials were conducted.

### 4D Label‐Free Quantitative Proteomics and Protein Pathway Analysis

The MSGs from wild‐type and *Draper* mutant groups were dissected at the prepupal stage and frozen in liquid nitrogen for proteomic analyses. Each group underwent three biological replications. GO analysis and KEGG analysis of DEPs were performed using the online platform of Majorbio Cloud Platform (www.majorbio.com) and bioinformatics (https://www.bioinformatics.com.cn). Heatmap was plotted by TBtools and edited by Adobe Photoshop CC 2019.

### Western Blotting

The standard western blotting procedure was carried out using antibodies targeting Draper (produced in our laboratory; 1:1 000), ATG8 (1:2000), ICE (produced in our laboratory; 1:2000), SQSTM1 (ABclonal Technology, A18679; 1:2000), ATG3 (ABclonal Technology, A5809; 1:2000), anti‐His (Proteintech Group, Inc, 66005‐1‐Ig; 1:2000), anti‐Flag (Hangzhou Huaan Biotechnology, M1403‐2; 1:2000) and Tubulin (1: 10 000). The Draper antibody was created in our library. A sequence (166 aa) of Draper was amplified by PCR using cDNA of the egg as a template with the primers shown in Table S1 (Supporting Information). The PCR fragments were integrated into the Nde I and Hind III sites within the prokaryotic expression vector pCold I (TaKaRa, 3361). *Escherichia coli* strain BL21 was transfected with the recombinant plasmid pCold I‐Draper. The shortened Draper protein underwent purification and was used to generate antiserum in rabbits. The polyclonal antibody against ATG8 was generously provided by Professor Wei Yu (Zhejiang Sci‐Tech University, PR China). The primary antibodies for ICE and Tubulin were prepared in our laboratory.^[^ ^71^ ^]^

Silkworm MSG and BmN cells were harvested and rinsed with cold PBS, and subsequently homogenized in ice‐cold RIPA lysate (FDbio Science Biotech Co. Ltd., FD009) (RIPA: PMSF = 100: 1) for 30 min. The protein content in the supernatant was measured with the bicinchoninic acid (BCA) assay. Proteins underwent separation using sodium dodecyl sulfate‐polyacrylamide gel electrophoresis (SDS‐PAGE), and subsequently transferred onto PVDF membranes (Millipore, IPVH00010). The membranes were blocked with 5% non‐fat milk and treated overnight with appropriate primary antibodies. Following incubation with a secondary antibody (DingGuo ChangSheng Biotechnology, IH‐0011/IH‐0031; 1:4 000) for 2 h, the signal was detected using ECL Plus Kit (FDbio Science Biotech Co. Ltd., FD8030).

### Plasmid Construction and Transfection

Cloned cDNAs of Draper (XM_03 801 3085.1) and ATG3 (NM_0 011 42489.1) from *B. mori* were inserted into the pIZ/V5 overexpression vector fused with His tag and Flag tag, respectively. BmN cells were transfected with plasmids containing Draper or ATG3 using Lipo8000 (Beyotime, C0533) following the manufacturer's instructions.

### Co‐IP Assay

To verify the interaction between ATG3 and Draper, in vitro Co‐IP experiments were conducted using BmN cells. BmN cells overexpressing different forms of free His and Draper–His were collected and lysed in cell lysis buffer for Western and IP (Beyotime Biotechnology, P0013) supplemented with a protease inhibitor cocktail (Roche China Holding Ltd., COEDTAF‐RO). Cells expressing free His served as a negative control. Following lysis, 10% of the supernatants were preserved as input controls. Lysates were then incubated with anti‐His magnetic beads (MedChemExpress, HY‐K0209) overnight at 4 °C, followed by four washes and boiling in sodium dodecyl sulfate loading buffer. Western blotting was performed after protein separation using sodium dodecyl sulfate‐polyacrylamide gel electrophoresis and transfer to a polyvinylidene difluoride membrane. An anti‐His antibody (1:4 000) was used to detect His‐tagged proteins. ATG3 antibody was used to identify Co‐IP with Draper.

### LC‐MS/MS Analysis

IP protein samples were separated using SDS‐PAGE until the dye front migrated to the bottom of the gel. Following staining with Coomassie Brilliant Blue, the entire strip was cut out and analyzed using a Q Exactive mass spectrometer through LC‐MS/MS.

### Statistical Analysis

The experimental data were analyzed via Student's t‐test using Prism 9.0.0 software (GraphPad Software). Statistically significant results were denoted by *p*‐value < 0.05, with symbols indicating the level of significance: ^*^ *p* < 0.05, ^**^ *p* < 0.01, ^***^ *p* < 0.001, ^****^ *p* < 0.0001, and ns, *p* > 0.05. Each data point was determined by averaging at least three independent experiments. The data represent the mean ± standard error of the mean.

### Availability of Data and Material

All data are available in the manuscript and in the Supplementary Information. The 4D label‐free quantitative proteomics of MSG in wild‐type and *Draper* mutant groups has been submitted to the Integrated Proteome Resources under the ProteomeXchange ID: PXD051512.

## Author Contributions

S.Z. and H.W. conceived the study conception and design. S.Z., Y.L., Y.J., W.J., Y.H., Y.L., Y.L., Z.H., and H.W. performed material preparation, data collection, and analysis. Y.X. and H.W. contributed to the supervision of the study. S.Z., Y.L., and H.W. wrote the manuscript draft. All authors read and approved the final manuscript.

## Conflict of Interest

The authors declare no conflict of interest.

## Supporting information



Supporting Information

Supporting Information

## Data Availability

The data that support the findings of this study are available in the supplementary material of this article.

## References

[1] I. Dikic , Annu. Rev. Biochem. 2017, 86, 193. 28460188 10.1146/annurev-biochem-061516-044908

[2] D. Denton , S. Nicolson , S. Kumar , Cell Death Differ. 2012, 19, 87. 22052193 10.1038/cdd.2011.146 PMC3252836

[3] H. Zhang , E. H. Baehrecke , Trends Cell Biol. 2015, 25, 376. 25862458 10.1016/j.tcb.2015.03.001 PMC4475674

[4] T. P. Neufeld , E. H. Baehrecke , Autophagy 2008, 4, 557. 18319640 10.4161/auto.5782 PMC2749667

[5] D. L. Berry , E. H. Baehrecke , Cell 2007, 131, 1137. 18083103 10.1016/j.cell.2007.10.048 PMC2180345

[6] W. Wu , M. Luo , K. Li , Y. Dai , H. Yi , Y. Zhong , Y. Cao , G. Tettamanti , L. Tian , Autophagy 2021, 17, 512. 32013726 10.1080/15548627.2020.1725376 PMC8007145

[7] R. Arya , K. White , Semin. Cell Dev. Biol. 2015, 39, 12. 25668151 10.1016/j.semcdb.2015.02.001 PMC4410075

[8] D. Denton , B. Shravage , R. Simin , E. H. Baehrecke , S. Kumar , Autophagy 2010, 6, 163. 20009534 10.4161/auto.6.1.10601 PMC2819273

[9] D. Denton , B. Shravage , R. Simin , K. Mills , D. L. Berry , E. H. Baehrecke , S. Kumar , Curr. Biol. 2009, 19, 1741. 19818615 10.1016/j.cub.2009.08.042 PMC2783269

[10] K. Mills , T. Daish , K. F. Harvey , C. M. Pfleger , I. K. Hariharan , S. Kumar , J. Cell Biol. 2006, 172, 809. 16533943 10.1083/jcb.200512126 PMC2063725

[11] T. J. Daish , K. Mills , S. Kumar , Dev. Cell 2004, 7, 909. 15572132 10.1016/j.devcel.2004.09.018

[12] D. Romanelli , M. Casartelli , S. Cappellozza , M. de Eguileor , G. Tettamanti , Sci. Rep. 2016, 6, 32939. 27609527 10.1038/srep32939 PMC5016986

[13] A. Montali , D. Romanelli , S. Cappellozza , A. Grimaldi , M. de Eguileor , G. Tettamanti , Arthropod Struct. Dev. 2017, 46, 518. 28549564 10.1016/j.asd.2017.05.003

[14] D. Denton , S. Kumar , Cell Death Differ. 2019, 26, 605. 30568239 10.1038/s41418-018-0252-y PMC6460387

[15] N. Mizushima , M. Komatsu , Cell 2011, 147, 728. 22078875 10.1016/j.cell.2011.10.026

[16] L. Galluzzi , E. H. Baehrecke , A. Ballabio , P. Boya , J. M. Bravo‐San Pedro , F. Cecconi , EMBO J. 2017, 36, 1811.28596378

[17] S. Fulda , D. Kögel , Oncogene 2015, 34, 5105. 25619832 10.1038/onc.2014.458

[18] D. Denton , T. Xu , S. Dayan , S. Nicolson , S. Kumar , Cell Death Differ. 2019, 26, 763. 29959404 10.1038/s41418-018-0154-z PMC6460390

[19] D. Denton , M. T. Aung‐Htut , N. Lorensuhewa , S. Nicolson , W. Zhu , K. Mills , D. Cakouros , A. Bergmann , S. Kumar , Nat. Commun. 2013, 4, 2916. 24336022 10.1038/ncomms3916 PMC3973156

[20] K. Tracy , P. D. Velentzas , E. H. Baehrecke , EMBO Rep. 2016, 17, 110. 26598552 10.15252/embr.201541283 PMC4718410

[21] I. Callebaut , V. Mignotte , M. Souchet , J. P. Mornon , Biochem. Biophys. Res. Commun. 2003, 300, 619. 12507493 10.1016/s0006-291x(02)02904-2

[22] J. Manaka , T. Kuraishi , A. Shiratsuchi , Y. Nakai , H. Higashida , P. Henson , Y. Nakanishi , J. Biol. Chem. 2004, 279, 48466. 15342648 10.1074/jbc.M408597200

[23] L. Cuttell , A. Vaughan , E. Silva , C. J. Escaron , M. Lavine , E. Van Goethem , J. P. Eid , M. Quirin , N. C. Franc , Cell 2008, 135, 524. 18984163 10.1016/j.cell.2008.08.033

[24] E. Kurant , S. Axelrod , D. Leaman , U. Gaul , Cell 2008, 133, 498. 18455990 10.1016/j.cell.2008.02.052 PMC2730188

[25] J. S. Ziegenfuss , R. Biswas , M. A. Avery , K. Hong , A. E. Sheehan , Y. G. Yeung , E. R. Stanley , M. R. Freeman , Nature 2008, 453, 935. 18432193 10.1038/nature06901 PMC2493287

[26] C. K. McPhee , M. A. Logan , M. R. Freeman , E. H. Baehrecke , Nature 2010, 465, 1093. 20577216 10.1038/nature09127 PMC2892814

[27] L. Lin , F. Rodrigues , C. Kary , A. Contet , M. Logan , R. H. G. Baxter , W. Wood , E. H. Baehrecke , Cell 2017, 170, 158. 28666117 10.1016/j.cell.2017.06.018 PMC5533186

[28] X. Wang , P. Zhao , Y. Li , Q. Yi , S. Ma , K. Xie , H. Chen , Q. Xia , Biomacromolecules 2015, 16, 3119. 26302212 10.1021/acs.biomac.5b00724

[29] E. Goncu , O. Parlak , Autophagy 2008, 4, 1069. 18838861 10.4161/auto.6953

[30] E. Kurucz , R. Markus , J. Zsamboki , K. Folkl‐Medzihradszky , Z. Darula , P. Vilmos , A. Udvardy , I. Krausz , T. Lukacsovich , E. Gateff , C. J. Zettervall , D. Hultmark , I. Ando , Curr. Biol. 2007, 17, 649. 17363253 10.1016/j.cub.2007.02.041

[31] T. T. Tung , K. Nagaosa , Y. Fujita , A. Kita , H. Mori , R. Okada , S. Nonaka , Y. Nakanishi , J. Biochem. 2013, 153, 483. 23420848 10.1093/jb/mvt014

[32] Y. Ichimura , T. Kirisako , T. Takao , Y. Satomi , Y. Shimonishi , N. Ishihara , N. Mizushima , I. Tanida , E. Kominami , M. Ohsumi , T. Noda , Y. Ohsumi , Nature 2000, 408, 488. 11100732 10.1038/35044114

[33] S. Makino , R. Hamajima , A. Saito , M. Tomizaki , A. Iwamoto , M. Kobayashi , H. Yamada , M. Ikeda , Dev. Comp. Immunol. 2018, 84, 133. 29448034 10.1016/j.dci.2018.02.009

[34] R. H. Chen , Y. H. Chen , T. Y. Huang , J. Biomed. Sci. 2019, 26, 80. 31630678 10.1186/s12929-019-0569-y PMC6802350

[35] P. E. Cockram , M. Kist , S. Prakash , S. H. Chen , I. E. Wertz , D. Vucic , Cell Death Differ. 2021, 28, 591. 33432113 10.1038/s41418-020-00708-5 PMC7798376

[36] Y. Jiang , S. Su , Y. Zhang , J. Qian , P. Liu , Oncogene 2019, 38, 3989. 30705402 10.1038/s41388-019-0713-x PMC6621562

[37] A. Martínez‐Férriz , A. Ferrando , A. Fathinajafabadi , R. Farràs , Semin. Cell Dev. Biol. 2022, 132, 146. 34952788 10.1016/j.semcdb.2021.12.009

[38] J. W. Locasale , L. C. Cantley , Cell Metab. 2011, 14, 443. 21982705 10.1016/j.cmet.2011.07.014 PMC3196640

[39] L. Herhaus , I. Dikic , Cell Res. 2018, 28, 389. 29572488 10.1038/s41422-018-0030-x PMC5939040

[40] M. Tsukada , Y. Ohsumi , FEBS Lett. 1993, 333, 169. 8224160 10.1016/0014-5793(93)80398-e

[41] F. Müller , C. Ádori , M. Sass , Eur. J. Cell Biol. 2004, 83, 67. 15146978 10.1078/0171-9335-00359

[42] Y. B. Li , T. Yang , J. X. Wang , X. F. Zhao , Front Endocrinol. (Lausanne) 2018, 9, 28. 29467720 10.3389/fendo.2018.00028 PMC5808327

[43] G. Tettamanti , M. Casartelli , Philos Trans R Soc Lond B Biol Sci 2019, 374, 20190065. 31438818 10.1098/rstb.2019.0065 PMC6711292

[44] T. Xu , X. Jiang , D. Denton , S. Kumar , Cell Death Differ. 2020, 27, 1. 31745213 10.1038/s41418-019-0456-9 PMC7205961

[45] Q. Xia , S. Li , Q. Feng , Annu. Rev. Entomol. 2014, 59, 513. 24160415 10.1146/annurev-ento-011613-161940

[46] Z. Li , L. You , B. Zeng , L. Ling , J. Xu , X. Chen , Z. Zhang , S. R. Palli , Y. Huang , A. Tan , Proc. Biol. Sci. 2015, 282, 20150513. 26041352 10.1098/rspb.2015.0513 PMC4590451

[47] S. Nicolson , D. Denton , S. Kumar , Int. J. Dev. Biol. 2015, 59, 23. 26374522 10.1387/ijdb.150055sk

[48] Y. Q. Di , X. L. Han , X. L. Kang , D. Wang , C. H. Chen , J. X. Wang , X. F. Zhao , Autophagy 2021, 17, 1170. 32324083 10.1080/15548627.2020.1752497 PMC8143247

[49] E. Franzetti , Z. J. Huang , Y. X. Shi , K. Xie , X. J. Deng , J. P. Li , Q. R. Li , W. Y. Yang , W. N. Zeng , M. Casartelli , H. M. Deng , S. Cappellozza , A. Grimaldi , Q. Xia , Q. Feng , Y. Cao , G. Tettamanti , Apoptosis 2012, 17, 305. 22127643 10.1007/s10495-011-0675-0

[50] J. M. MacDonald , M. G. Beach , E. Porpiglia , A. E. Sheehan , R. J. Watts , M. R. Freeman , Neuron 2006, 50, 869. 16772169 10.1016/j.neuron.2006.04.028

[51] S. B. Serizier , J. S. Peterson , K. McCall , J. Cell Sci. 2022, 135, jcs250134. 36177600 10.1242/jcs.250134 PMC10658789

[52] R. Hilu‐Dadia , K. Hakim‐Mishnaevski , F. Levy‐Adam , E. Kurant , Glia 2018, 66, 1520. 29520845 10.1002/glia.23322

[53] S. M. Hill , L. Wrobel , D. C. Rubinsztein , Cell Death Differ. 2019, 26, 617. 30546075 10.1038/s41418-018-0254-9 PMC6460389

[54] R. Nakano , M. Iwamura , A. Obikawa , Y. Togane , Y. Hara , T. Fukuhara , M. Tomaru , T. Takano‐Shimizu , H. Tsujimura , Dev. Biol. 2019, 453, 68. 31063730 10.1016/j.ydbio.2019.05.003

[55] D. Fang , H. Xie , T. Hu , H. Shan , M. Li , Front. Cell. Dev. Biol. 2021, 9, 685625. 34235149 10.3389/fcell.2021.685625 PMC8255673

[56] Y. Ye , E. R. Tyndall , V. Bui , M. C. Bewley , G. Wang , X. Hong , Y. Shen , J. M. Flanagan , H. G. Wang , F. Tian , Nat. Commun. 2023, 14, 5503. 37679347 10.1038/s41467-023-41243-4 PMC10485044

[57] B. H. Yoo , A. Zagryazhskaya , Y. Li , A. Koomson , I. A. Khan , T. Sasazuki , S. Shirasawa , K. V. Rosen , Autophagy 2015, 11, 1230. 26061804 10.1080/15548627.2015.1056968 PMC4590629

[58] J. S. Lee , Q. Li , J. Y. Lee , S. H. Lee , J. H. Jeong , H. R. Lee , H. Chang , F. C. Zhou , S. J. Gao , C. Liang , J. U. Jung , Nat. Cell Biol. 2009, 11, 1355. 19838173 10.1038/ncb1980 PMC2802862

[59] S. Han , Y. Wang , X. Zheng , Q. Jia , J. Zhao , F. Bai , Y. Hong , Y. Liu , The Plant Cell 2015, 27, 1316. 25829441 10.1105/tpc.114.134692 PMC4558687

[60] S. Liu , H. Tian , Y. Xu , H. Wang , Cell. Mol. Life Sci. 2023, 80, 331. 37870631 10.1007/s00018-023-04996-1 PMC11071706

[61] Y. Li , Z. Liu , C. Liu , Z. Shi , L. Pang , C. Chen , Y. Chen , R. Pan , W. Zhou , X. X. Chen , A. Rokas , J. Huang , X. X. Shen , Cell 2022, 185, P2975. 10.1016/j.cell.2022.06.014 PMC9357157 35853453

[62] Z. Hao , Q. Lu , Y. Zhou , Y. Liang , Y. Gao , H. Ma , Y. Xu , H. Wang , Pestic. Biochem. Physiol. 2023, 196, 105610. 37945249 10.1016/j.pestbp.2023.105610

[63] H. Tian , S.‐Q. Liu , W.‐H. Jing , Z.‐H. Hao , Y.‐H. Li , Z.‐H. Lu , Z.‐K. Ding , S.‐L. Huang , Y.‐S. Xu , H.‐B. Wang , Arch. Insect Biochem. Physiol. 2023, 112, 21995. 10.1002/arch.21995 36575612

[64] Y. Wang , Z. Li , J. Xu , B. Zeng , L. Ling , L. You , Y. Chen , Y. Huang , A. Tan , Cell Res. 2013, 23, 1414. 24165890 10.1038/cr.2013.146 PMC3847576

[65] J. D. Sander , M. L. Maeder , D. Reyon , D. F. Voytas , J. K. Joung , D. Dobbs , Nucleic Acids Res. 2010, 38, W462. 20435679 10.1093/nar/gkq319 PMC2896148

[66] Y. Gao , Y. C. Liu , S. Z. Jia , Y. T. Liang , Y. Tang , Y. S. Xu , H. Kawasaki , H. B. Wang , PLoS Genet. 2020, 16, 1008980. 10.1371/journal.pgen.1008980 PMC7544146 32986708

[67] Z. Yu , M. Ren , Z. Wang , B. Zhang , Y. Rong , R. Jiao , G. Gao , Genetics 2013, 195, 289. 23833182 10.1534/genetics.113.153825 PMC3761309

[68] W. Wei , H. Xin , B. Roy , J. Dai , Y. Miao , G. Gao , PLoS One 2014, 9, 101210. 10.1371/journal.pone.0101210 PMC4094479 25013902

[69] X. Tang , X. Ye , X. Wang , S. Zhao , M. Wu , J. Ruan , B. Zhong , Sci. Rep. 2021, 11, 20980. 34697320 10.1038/s41598-021-00029-8 PMC8546084

[70] L. Xie , X. J. Song , Z. F. Liao , B. Wu , J. Yang , H. Zhang , J. Hong , Micron 2019, 120, 80. 30807983 10.1016/j.micron.2019.01.007

[71] Q. Lu , S. Xu , Z. Hao , Y. Li , Y. Huang , S. Ying , W. Jing , S. Zou , Y. Xu , H. Wang , J. Hazard. Mater. 2023, 458, 131997. 37423129 10.1016/j.jhazmat.2023.131997

