# Supplementary material for: Draper‐ATG3 Interaction Positively Regulates Autophagy to Mediate Silk Gland Degradation in Bombyx mori
Source: Adv Sci (Weinh). 2025 Sep 15;12(45):e04664. doi: 10.1002/advs.202504664 (PMC12677660; doi:10.1002/advs.202504664)
Supplement: Supplementary file 1 — Supporting Information [file ADVS-12-e04664-s001.docx]

**Supplement material**

## ****Draper**–**ATG3 Interaction Positively Regulates Autophagy to Mediate Silk Gland Degradation in *Bombyx mori*****

Shiyu Zou^1^, Yuhan Luo^1^, Yue Jin^1^, Wenhui Jing^1^, Yuxin Huang^1^, Yanting Liang^1^, Yinghui Li^1^, Zhihua Hao^1^, Yusong Xu^1^, Huabing Wang^1,^**^*^**

^1^College of Animal Sciences, Zhejiang University, Hangzhou, China

**^*^**Correspondence: Huabing Wang, Email: [wanghb@zju.edu.cn](mailto:wanghb@zju.edu.cn)

**
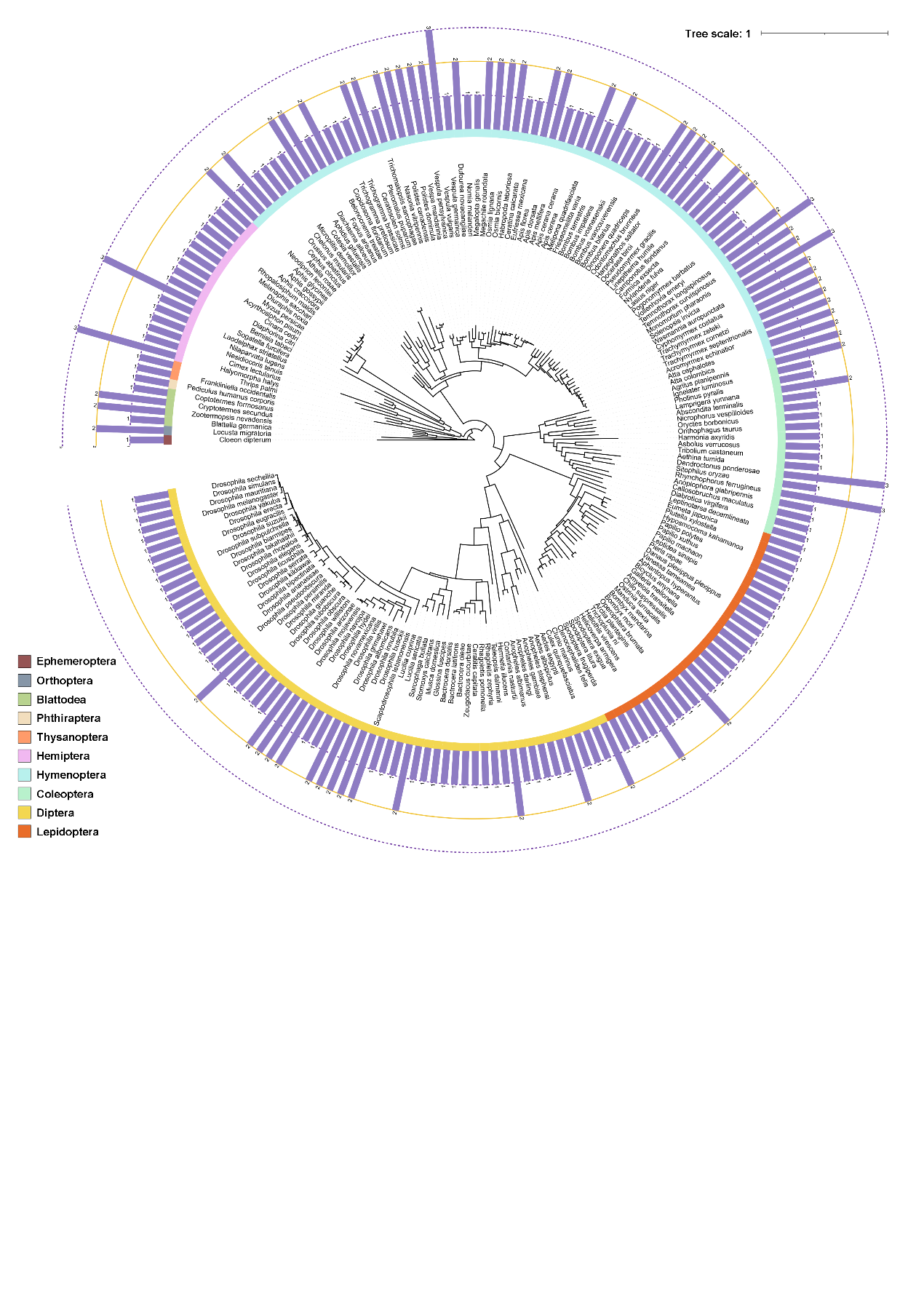
Fig. S1** Gene copy numbers of *Draper* in 202 insects. 277 *Draper* homologous genes were retrieved from 202 insect species and visualized in the phylogenetic tree of these insects. The quantity of *Draper* genes is depicted by the purple bars. Each circle of scale represents an increase of one gene. Different colors indicate different insect orders.


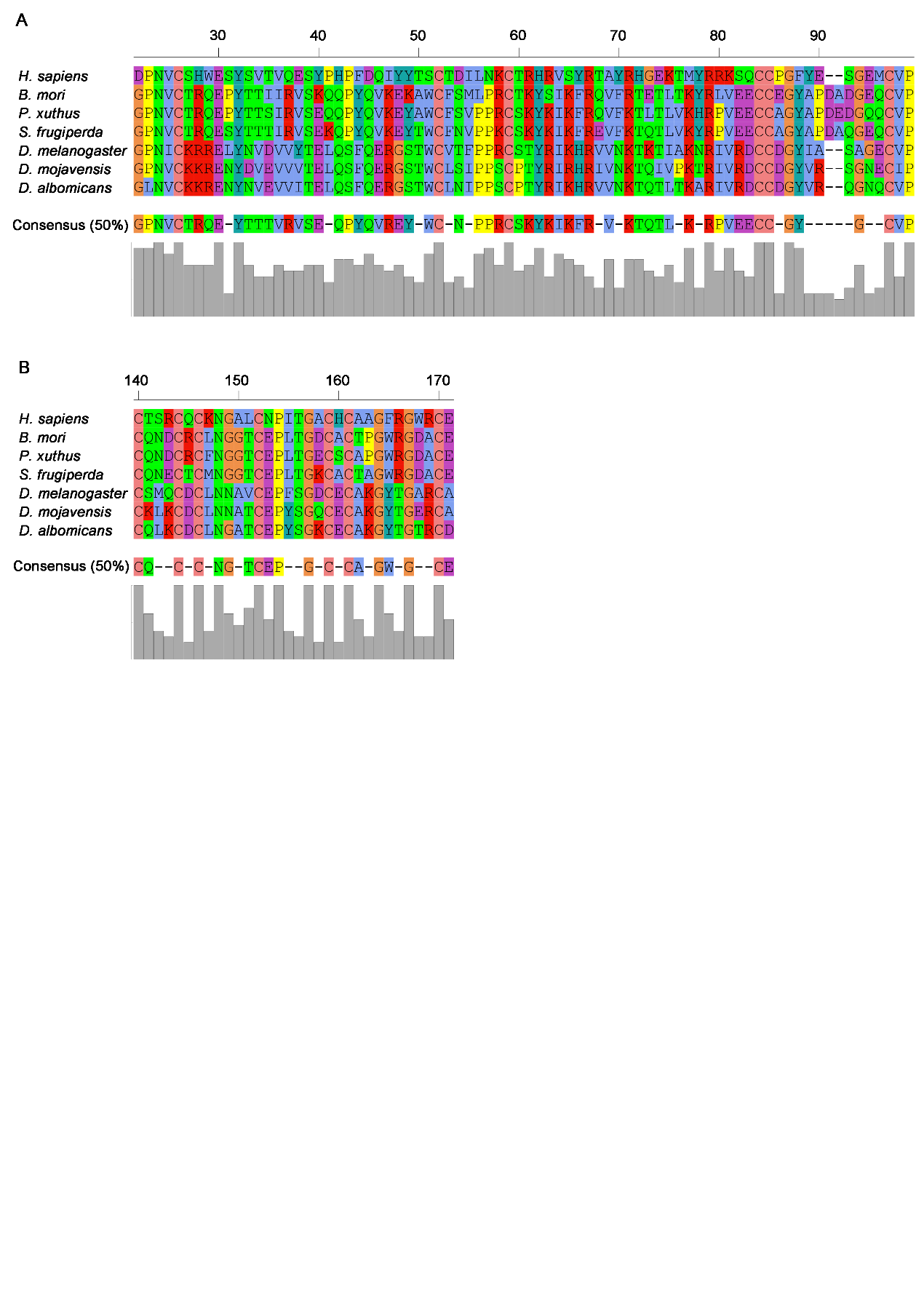


**Fig. S2** Amino acid sequence comparison of conserved domains of Draper. (**A**) Alignments of amino acid sequences of Draper proteins from seven species in the EMI domain region. (**B**) Alignments of amino acid sequences of Draper proteins from seven species in the first EGF-like domain region.

**
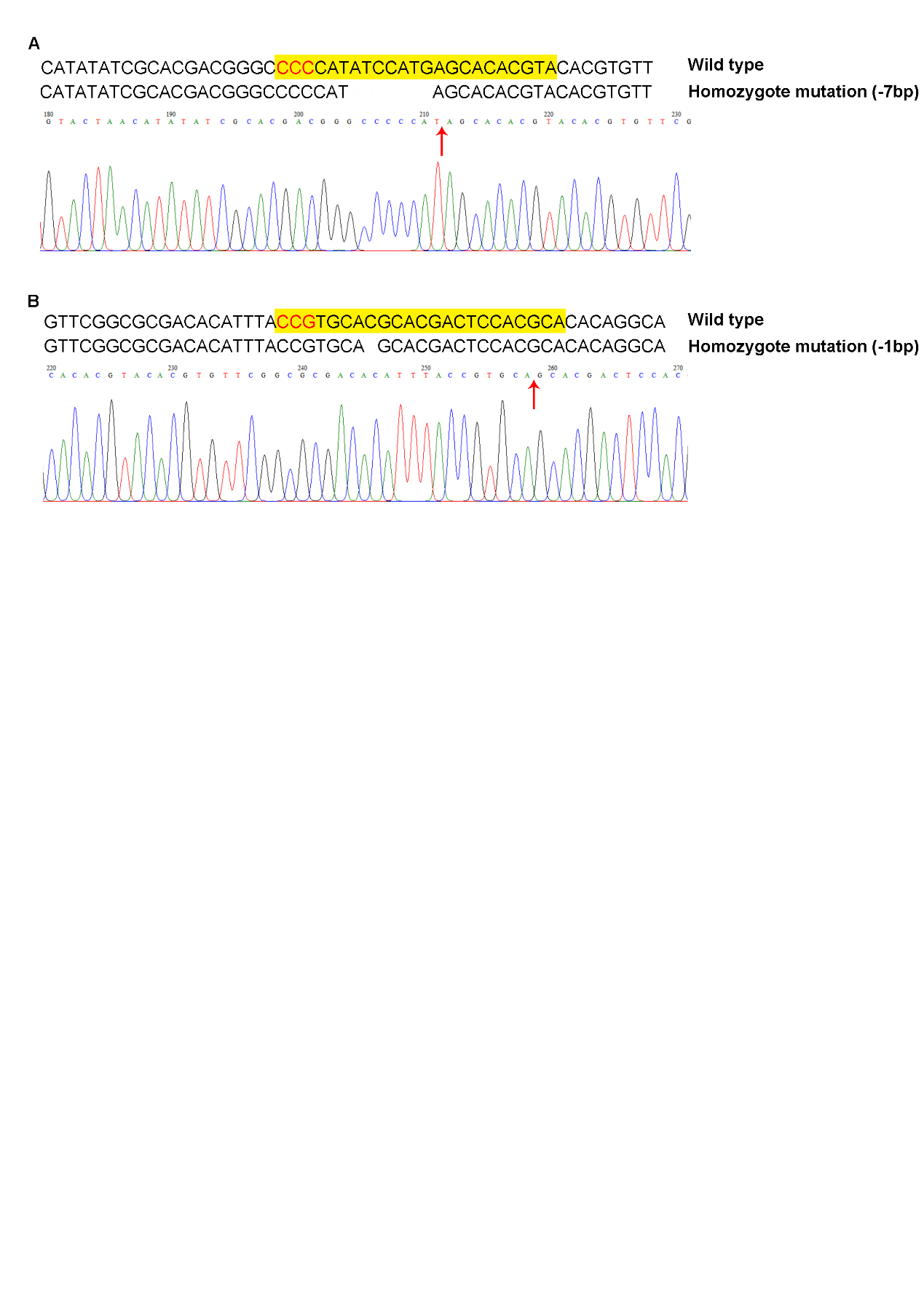
Fig. S3** Mutation types of chromosomal deletions and sequencing peak maps. The mutant genotype and sequencing peak diagram of -7bp deletion (**A**) and -1bp deletion (**B**). Yellow area represents the sgRNA targets, red font represents the PAM sequence, and red arrow represents the mutation position in the peak diagram.

**
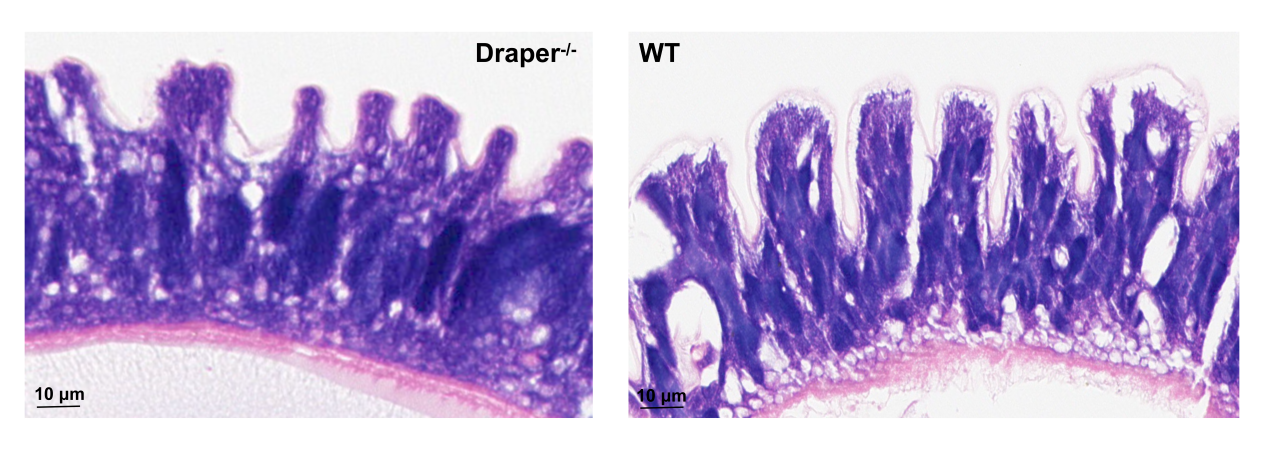
**

**Fig. S4** **Histomorphological images of middle MSG at the prepupal stage.** HE staining of the cross section shows the tissue histology middle MSG at the prepupal stage.


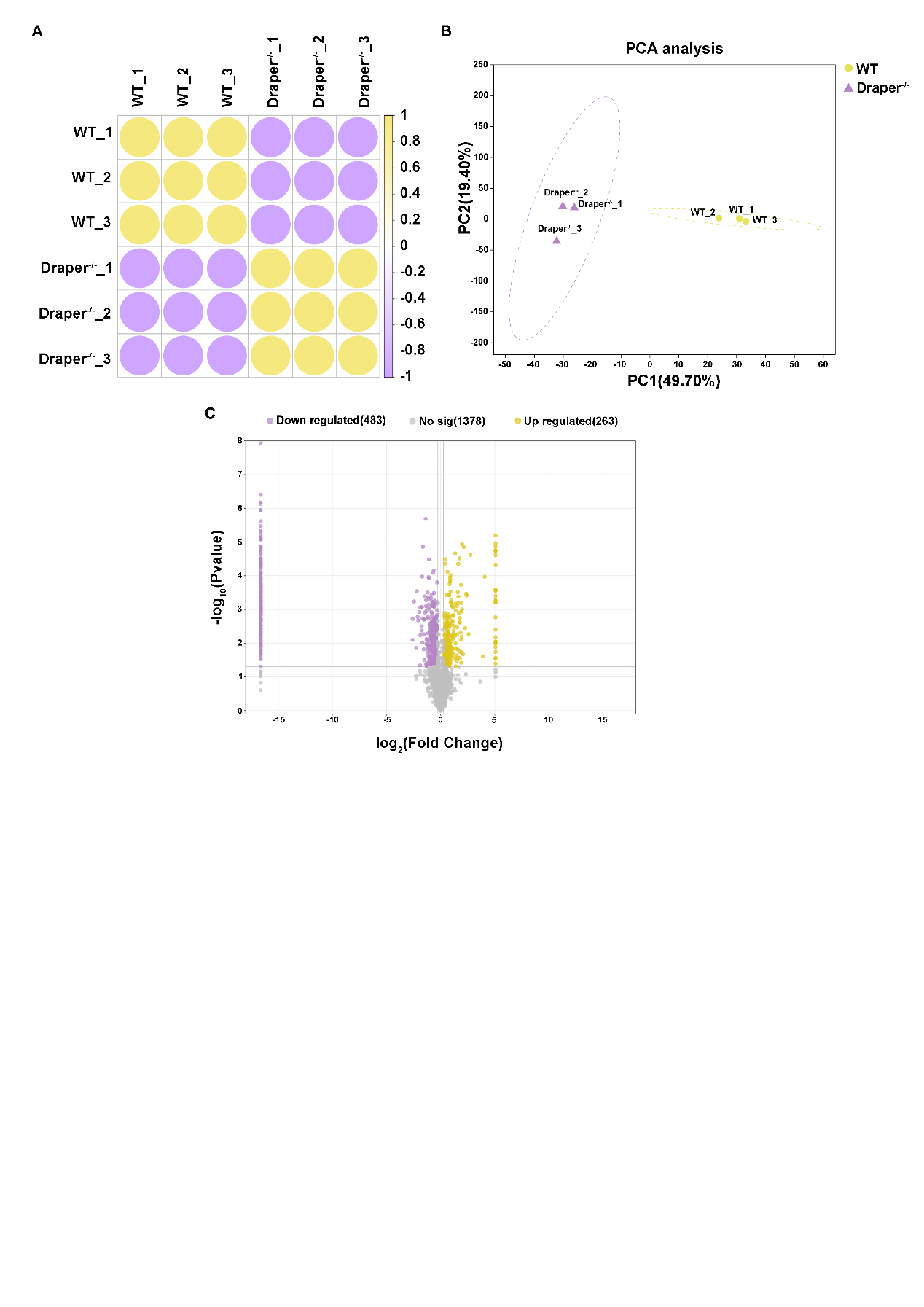


**Fig. S5** Proteomic sample identification. (**A**) Pearson correlation analysis of wild-type and *Draper* mutant groups. (**B**) Principal component analysis of wild-type and *Draper* mutant groups. (**C**) Volcano plot for DEPs. Yellow and purple dots indicate upregulated and downregulated DEPs, respectively. DEPs were identified with |log2 (fold change)| > 1.2 or < 0.83, and p < 0.05.

**
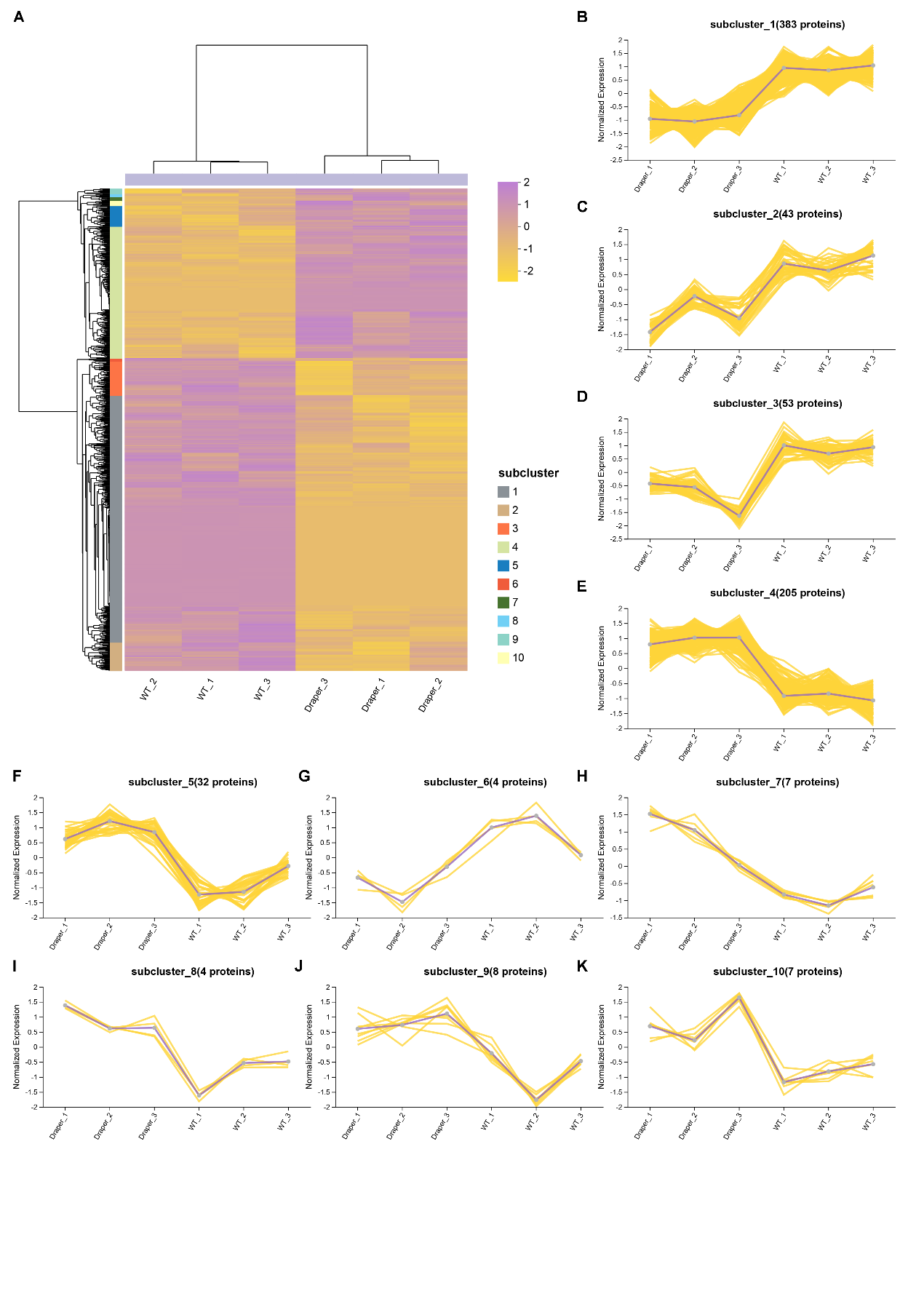
Fig. S6** Expression pattern clustering. (**A**) Heat map of clustering of DEPs. Each column in the graph represents a sample and each row represents a protein. Different colors in the graph indicate the relative expression size of the protein in the group of samples. On the left side is a dendrogram of the protein clusters. The closer distance between two protein branches, the closer their expression. Above is a dendrogram of sample clustering. The closer distance between two sample branches, the closer the expression patterns of all proteins in these two samples. (**B-K**) Subcluster trend plot. A line graph of expression trends for each subcluster in (**A**). The horizontal coordinates are for each comparative sample group, and the vertical coordinates are the expression of the proteins in that group of samples. Each yellow line in the graph represents one protein, and the purple line represents the average of the expression of all proteins in that subcluster.

**
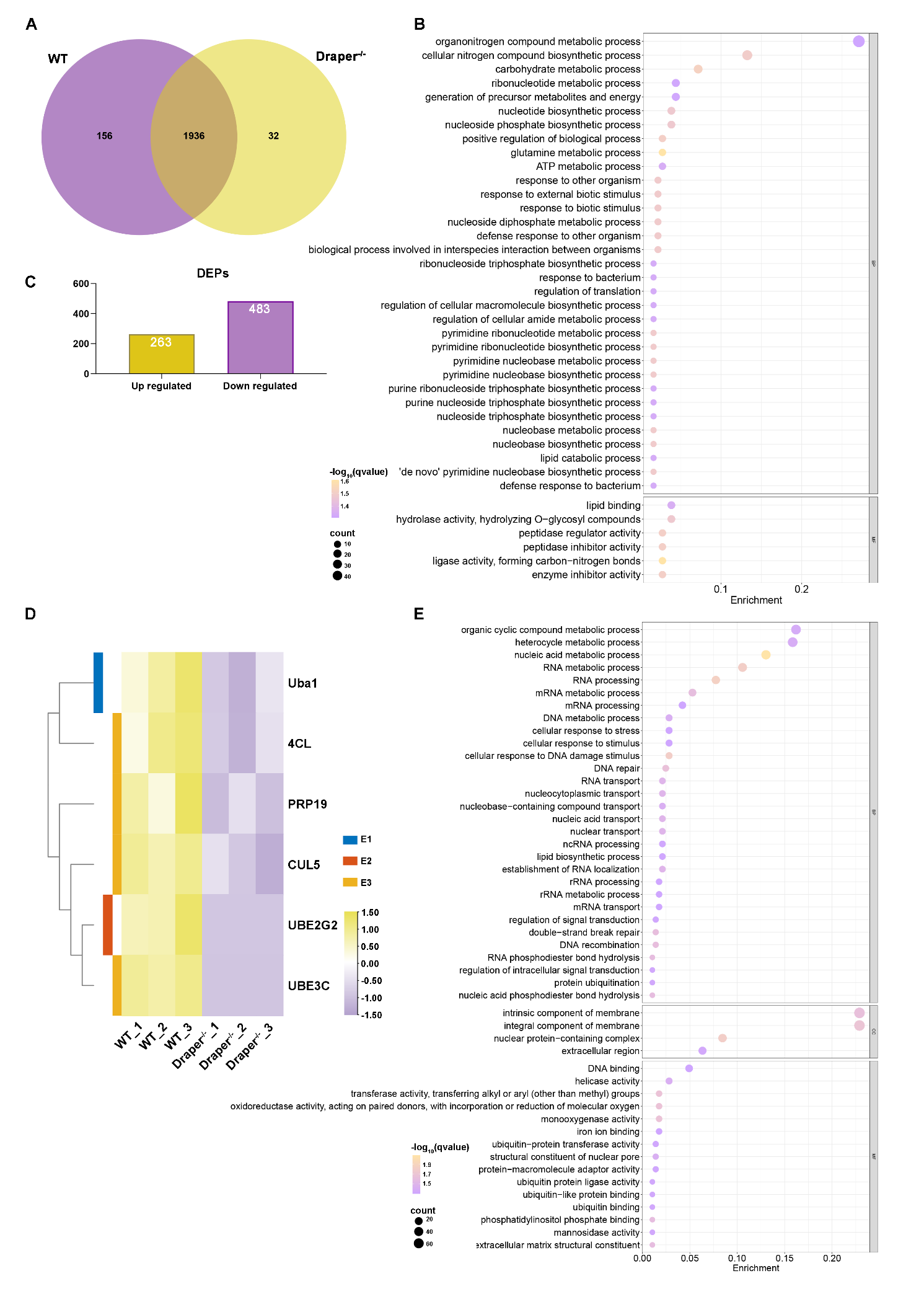
**

**Fig. S7** Identification and GO analysis of the DEPs observed between the wild-type and *Draper* mutant groups. (**A**) Venn diagram of proteins identified in the wild-type and *Draper* mutant groups. Purple and yellow squares represent the wild-type and *Draper* mutant groups, respectively. (**B,** **E**) GO enrichment analysis of (**B**) upregulated DEPs and (**E**) downregulated DEPs between the wild-type and *Draper* mutant groups. Different colors represent q-values. Point size indicates the quantity of proteins enriched in a pathway. (**C**) Number of upregulated and downregulate DEPs. (**D**) Heat map of ubiquitin-related proteins significantly regulated in the wild-type and *Draper* mutant groups. Different colors indicate the relative expression levels of proteins. Left side: dendrogram of protein clusters and classification annotations for proteins. The closer the distance between two protein branches, the more similar their expression. For protein classification annotations, different categories are shown in different colors.

**
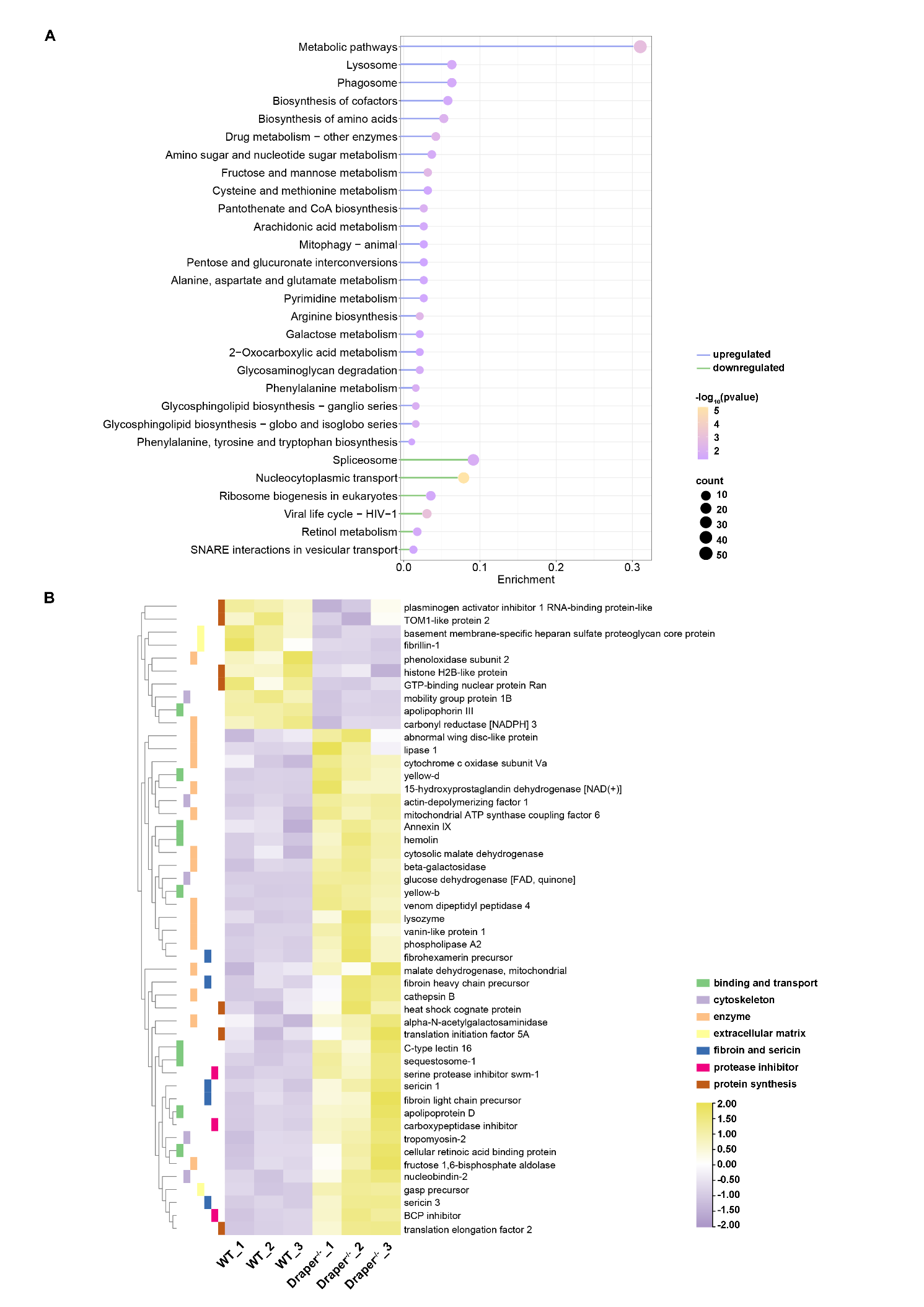
**

**Fig. S8** KEGG analysis of DEPs and the heatmap of silk-related proteins. (**A**) KEGG analysis of upregulated DEPs and downregulated DEPs between the wild-type and *Draper* mutant groups. Different colors present *p*-values. Point size indicates the quantity of proteins enriched in a pathway. Blue and green lines represent upregulated DEPs and downregulated DEPs enrichment pathways, respectively. (**B**) Heat map of silk-related proteins significantly regulated in the wild-type and *Draper* mutant groups. Different colors indicate the relative expression levels of proteins. Left side: dendrogram of protein clusters and classification annotations for proteins. The closer the distance between two protein branches, the more similar their expression. For protein classification annotations, different categories are shown in different colors.
